# Vitamin D and Gastric Cancer: A Systematic Review and Meta-Analysis

**DOI:** 10.3390/medsci14050562

**Published:** 2026-09-11

**Authors:** Roxana Pântea, Lorena Elena Meliț

**Affiliations:** 1 Faculty of Medicine, “George Emil Palade” University of Medicine, Pharmacy, Sciences and Technology of Târgu Mureș, Gheorghe Marinescu Street No. 38, 540139 Târgu Mureș, Romania; lorena.melit@umfst.ro or lory_chimista89@yahoo.com; 2 Department of Paediatrics II, “George Emil Palade” University of Medicine, Pharmacy, Sciences and Technology of Târgu Mureș, Gheorghe Marinescu Street No. 38, 540139 Târgu Mureș, Romania

**Keywords:** vitamin D, gastric cancer, 25-hydroxyvitamin D, vitamin D receptor, VDR polymorphisms, FokI polymorphism, meta-analysis

## Abstract

**Background/Objectives:** Gastric cancer is a major global health challenge with multifactorial aetiology. Vitamin D has been investigated for a potential role in gastric carcinogenesis, but evidence remains heterogeneous. This systematic review aimed to synthesise evidence on serum 25(OH)D, dietary vitamin D intake and VDR polymorphisms in relation to gastric cancer in adults and children. **Methods:** A systematic search following PRISMA 2020 standards was conducted in PubMed, Web of Science, Scopus and Lens.org for studies published from January 2007 to January 2026. Observational studies evaluating vitamin D exposure or VDR polymorphisms in relation to gastric cancer were eligible. Risk of bias was assessed using established instruments. Where comparable data were available, random-effects meta-analyses were performed, with REML estimation and Knapp–Hartung adjustment. **Results:** Twenty-four studies met the inclusion criteria, and no eligible paediatric studies were identified. Four studies evaluating serum 25(OH)D showed a non-significant tendency towards lower concentrations in gastric cancer patients (MD = −8.82 ng/mL, 95% CI −18.15 to 0.51). Five studies evaluating dietary vitamin D showed no significant association with gastric cancer risk (OR = 0.87, 95% CI 0.47–1.60). Five studies evaluating the VDR FokI polymorphism under the dominant model demonstrated increased gastric cancer susceptibility among variant carriers (OR = 1.44, 95% CI 1.03–2.01). **Conclusions:** Associations between vitamin D and gastric cancer varied by exposure type. Serum 25(OH)D and dietary intake showed no significant pooled associations, whereas VDR FokI was associated with increased gastric cancer susceptibility. The absence of paediatric evidence highlights an important knowledge gap.

## 1. Introduction

Gastric cancer (GC) remains a major global health challenge despite a gradual decline in incidence over recent decades. According to the latest GLOBOCAN estimates, GC ranks as the fifth most frequently diagnosed malignancy and the fifth leading cause of cancer-related mortality worldwide, accounting for 968,784 new cases and 660,175 deaths in 2022. The global distribution of GC is highly heterogeneous, with more than 70% of incident cases occurring in Asia, particularly Eastern Asia, whereas substantially lower incidence rates are observed in North America and Africa [1,2]. Although advances in endoscopic screening and multimodal treatment have improved outcomes in selected populations, the prognosis of advanced GC remains poor, with a 5-year overall survival below 30% in most countries due to late-stage diagnosis [3]. The development of gastric cancer is multifactorial, resulting from a complex interaction between lifestyle, environmental exposures, infectious agents, dietary habits and host genetic susceptibility. That being said, factors like tobacco smoking, excessive alcohol consumption, high dietary salt intake, low consumption of fruits and vegetables, red meat, processed meat, obesity, Epstein–Barr virus infection, autoimmune gastritis and hereditary cancer syndromes are directly implicated in gastric carcinogenesis [4,5,6]. Chronic Helicobacter pylori infection remains the strongest established risk factor and has been classified as a Group I carcinogen by the International Agency for Research on Cancer [7]. Nevertheless, these factors do not fully explain the considerable inter-individual variability in disease occurrence, prompting increasing interest in additional environmental and metabolic factors, including vitamin D.

Vitamin D is a fat-soluble secosteroid hormone traditionally recognised for its major role in calcium homeostasis and bone maintenance. Following cutaneous synthesis or dietary intake, vitamin D undergoes sequential hydroxylation in the liver and kidneys to produce the biologically active metabolite 1,25-dihydroxyvitamin D, which exerts its effects primarily through the vitamin D receptor (VDR), a ligand-activated nuclear transcription factor expressed in numerous tissues, including the gastrointestinal epithelium [8,9,10,11]. Beyond its classical endocrine functions, vitamin D has emerged as a pleiotropic regulator of cellular homeostasis, influencing cell proliferation, differentiation, apoptosis, oxidative stress, inflammation and immune responses through VDR-mediated genomic and non-genomic signalling pathways [10,11,12,13,14]. Experimental and translational evidence further suggests that vitamin D may contribute to the prevention of gastric carcinogenesis by modulating key molecular pathways involved in tumour initiation and progression, including Wnt/β-catenin and NF-κB signalling, while maintaining epithelial integrity and regulating the host immune response to *Helicobacter pylori*, thereby potentially attenuating the chronic inflammatory cascade that precedes intestinal metaplasia, dysplasia and gastric cancer development [14,15,16]. Despite compelling experimental evidence supporting the antitumor effects of vitamin D, epidemiological findings regarding its association with gastric cancer remain inconclusive [10,11]. Systematic reviews evaluating circulating 25-hydroxyvitamin D concentrations have reported heterogeneous results, with some meta-analyses suggesting an inverse association between vitamin D deficiency and gastric cancer risk or prognosis, whereas others concluded that the available evidence remains insufficient to establish a consistent relationship [17,18,19]. Likewise, evidence regarding dietary vitamin D intake and VDR gene polymorphisms remains inconsistent, with reported associations varying according to ethnicity, genetic model and study design [20,21]. The observed heterogeneity has been attributed to differences in population characteristics, ethnicity, methods used to assess vitamin D exposure, laboratory assays, dietary assessment tools, adjustment for confounding factors and underlying genetic susceptibility [21,22,23]. Although several systematic reviews and meta-analyses have investigated different aspects of vitamin D in gastric or gastrointestinal cancers, most evaluated only one or two dimensions of vitamin D exposure or were conducted before several recent observational studies became available, leaving the evidence fragmented. Furthermore, several observational studies investigating circulating vitamin D concentrations, dietary intake and VDR polymorphisms have been published in recent years, highlighting the need for an updated and comprehensive synthesis of the available evidence [21]. Therefore, the present systematic review aimed to comprehensively evaluate the association between vitamin D and gastric cancer by integrating evidence from observational studies investigating circulating vitamin D concentrations, dietary vitamin D intake and VDR gene polymorphisms. Additionally, a meta-analysis was performed whenever sufficient homogeneous data were available to quantitatively synthesise the existing evidence.

## 2. Materials and Methods

### 2.1. Study Design

This systematic review and meta-analysis were conducted in accordance with the Preferred Reporting Items for Systematic Reviews and Meta-Analyses (PRISMA) 2020 Statement [24,25]. The objective of this review was to evaluate the association between vitamin D and gastric cancer by integrating evidence regarding circulating vitamin D concentrations, dietary vitamin D intake and vitamin D receptor (VDR) gene polymorphisms. The review was not registered at PROSPERO.

### 2.2. Literature Search Strategy

A comprehensive literature search was performed in PubMed, Web of Science, Scopus and Lens.org to identify studies investigating the association between vitamin D and gastric cancer. The final literature search was conducted in January 2026, encompassing all relevant articles published from January 2007 up to the time of the search, using search strings that combined controlled vocabulary and free-text terms related to gastric cancer and vitamin D. The PubMed strategy included: (“Gastric Cancer” OR “Stomach Neoplasms”) AND (“Vitamin D” OR “25-hydroxyvitamin D” OR cholecalciferol OR “Vitamin D Receptor” OR “VDR polymorphisms”), while the complete strategies for all databases are provided in Supplementary File S1. Searches were limited to human studies published in English, without restrictions on geographical region or study setting. Reference lists of eligible studies and relevant reviews were also screened manually.

### 2.3. Eligibility Criteria

Eligibility criteria were defined according to the PECOS framework.

Studies involving paediatric or adult populations with gastric cancer, individuals at risk of gastric cancer, or healthy controls were considered eligible. Eligible exposures included circulating 25-hydroxyvitamin D [25(OH)D] concentrations, dietary vitamin D intake, and VDR gene polymorphisms. Comparators included healthy controls or alternative exposure categories, as reported by individual studies. The primary outcome was gastric cancer risk or occurrence, reported as ORs, RRs, HRs, or sufficient data for their calculation. Eligible study designs included observational case–control, cohort, and cross-sectional studies. Review articles and meta-analyses, editorials, letters, conference abstracts without full-text availability, case reports, animal and in vitro studies, duplicate publications, non-English articles, and studies not evaluating gastric cancer as an outcome were excluded.

### 2.4. Study Selection

All retrieved references were imported into Rayyan [26], where duplicate records were identified and removed before screening. Two reviewers independently screened titles and abstracts according to the predefined eligibility criteria. Full texts of potentially eligible studies were subsequently assessed independently. Any disagreements regarding study eligibility were resolved through discussion until consensus was reached.

**Figure 1:**
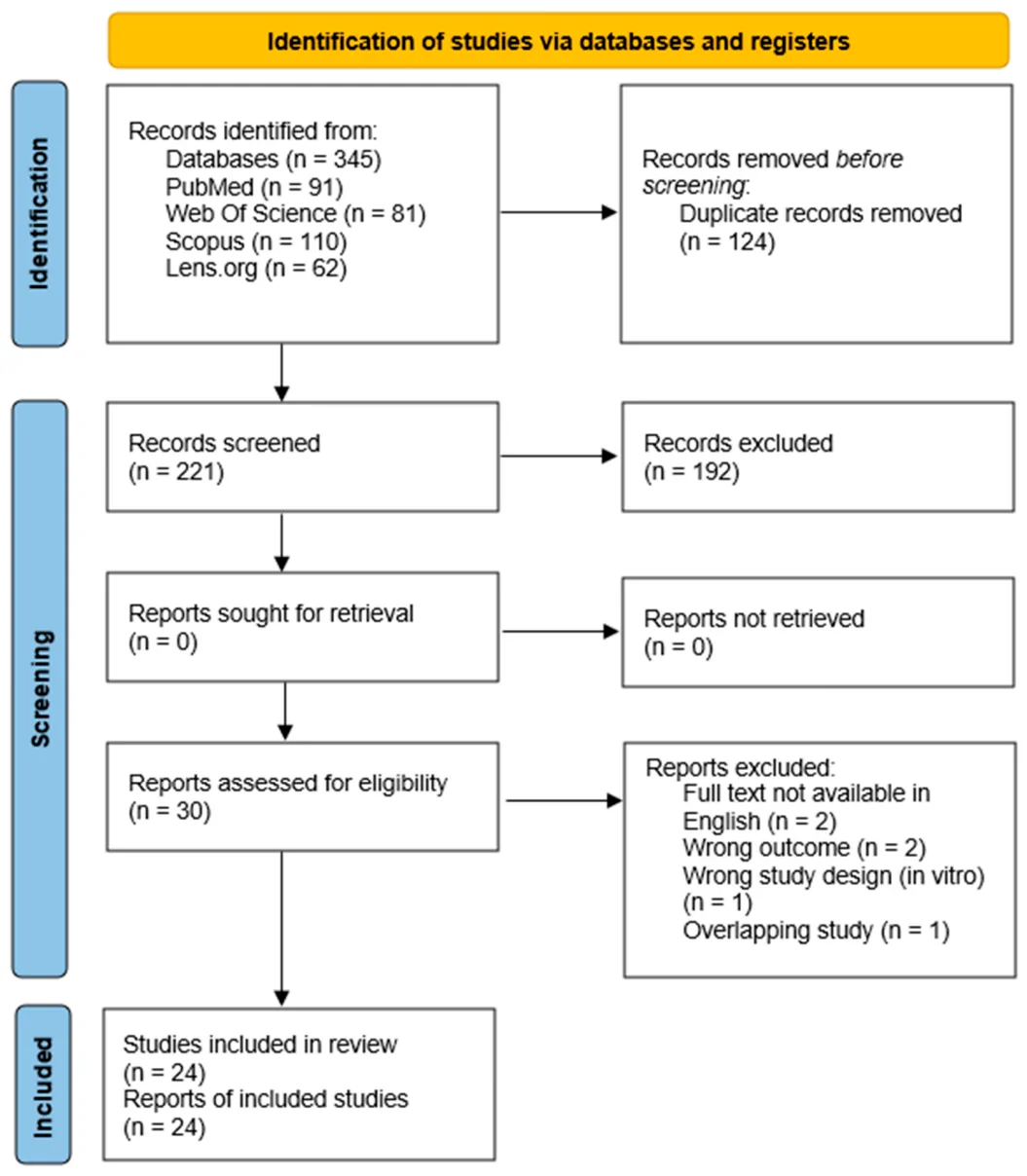
PRISMA flow diagram of the study selection process.

The literature search identified 345 records, of which 124 duplicate records were removed. The remaining 221 studies underwent title and abstract screening, resulting in 30 full-text articles assessed for eligibility. Six studies were excluded after full-text review because the full text was unavailable in English (*n* = 2), the reported outcome was not relevant to the review question (*n* = 2), the study used an in vitro experimental design (*n* = 1), or the study was a secondary pooled analysis with overlapping populations (*n* = 1). Ultimately, 24 studies fulfilled the eligibility criteria and were included in the systematic review. The study selection process is illustrated in the PRISMA flow diagram (Figure 1).

### 2.5. Data Extraction

Data extraction was performed independently by two reviewers using a standardised data extraction form, disagreements being resolved through discussion and consensus. The following information was collected from each eligible study: first author, publication year, country, study design, study population and sample size, type of vitamin D exposure (serum vitamin D concentration, dietary vitamin D intake or VDR polymorphism), exposure assessment method (e.g., serum assay, food-frequency questionnaire or genotyping technique), main outcome measures, effect estimates (OR, RR, HR or mean difference, as appropriate) with corresponding 95% confidence intervals, variables included in multivariable analyses when reported, and eligibility for qualitative and quantitative synthesis. Whenever multiple publications reported results from overlapping populations, only the study providing the most comprehensive dataset was included. No assumptions were made for missing data.

### 2.6. Risk of Bias Assessment

Methodological quality and risk of bias were evaluated independently by two reviewers using study-design-specific assessment tools.

Case–control and cohort studies were assessed using the Newcastle–Ottawa Scale (NOS) [27], cross-sectional studies were evaluated using the Appraisal Tool for Cross-Sectional Studies (AXIS) [28] and prognostic studies were assessed using the Quality In Prognosis Studies (QUIPS) tool [29].

### 2.7. Data Synthesis and Statistical Analysis

A qualitative synthesis was performed for all eligible studies. Whenever sufficient clinically and methodologically homogeneous studies reported comparable effect estimates, a quantitative synthesis (meta-analysis) was performed using R (version 4.6.1) with the metafor package (version 5.0-1) [30]. Random-effects models with the restricted maximum-likelihood (REML) estimator and Knapp–Hartung adjustment were applied because methodological and clinical heterogeneity among studies was anticipated. Mean differences (MDs) with 95% confidence intervals (95% CIs) were calculated for studies evaluating circulating vitamin D concentrations, whereas odds ratios (ORs) with corresponding 95% CIs were used for studies investigating dietary vitamin D intake and VDR gene polymorphisms. Statistical heterogeneity was assessed using Cochran’s Q test and quantified using the I^2^ statistic. Prediction intervals were calculated for all meta-analyses, and sensitivity analyses were performed using leave-one-out and influence analyses to evaluate the robustness of the pooled estimates and the influence of individual studies. Meta-analyses were conducted separately according to the investigated exposure, including circulating vitamin D concentrations, dietary vitamin D intake, and VDR gene polymorphisms.

## 3. Results

### 3.1. Study Characteristics

A total of 24 studies published between 2007 and 2025 were included in the qualitative synthesis (Table 1). Because several studies assessed more than one vitamin D-related exposure domain, these categories were not mutually exclusive. The studies were conducted across diverse geographic settings, including China, India, Iran, South Korea, Turkey, Jordan, Vietnam, Italy, Japan, and the United States, as well as multicenter cohorts including participants from China, Finland, the United States, and the international EXPAND trial population. Most studies used a case–control design, although prospective cohort, nested case–control, case–cohort, cross-sectional, retrospective biomarker, and prognostic cohort designs were also represented.

Ten studies investigated circulating serum 25(OH)D concentrations, eight evaluated dietary vitamin D intake, and nine examined VDR gene polymorphisms. Studies providing sufficiently homogeneous outcome measures contributed to the quantitative meta-analyses, whereas the remaining studies were synthesised narratively.

### 3.2. Risk of Bias Assessment

The methodological quality of the included studies was assessed using study design-specific risk-of-bias tools. Overall, the methodological quality of the studies included was considered acceptable. According to the NOS assessment, nine studies were classified as having a low risk of bias (8–9 stars), while the remaining observational studies were rated as having a moderate risk of bias (6–7 stars). There was no study classified as high risk of bias (<5 stars). Both cross-sectional studies had a good methodological quality with only minor reporting limitations according to the AXIS assessment. Likewise, the two prognostic cohort studies were judged to have a low risk of bias across all QUIPS domains. Detailed assessments are presented in Table 2, Table 3 and Table 4.

### 3.3. Serum Vitamin D and Gastric Cancer

#### 3.3.1. Narrative Synthesis

Ten studies investigated circulating serum or plasma 25(OH)D concentrations in relation to gastric cancer, including eight studies evaluating gastric cancer risk or prevalence and two studies assessing the prognostic significance of baseline vitamin D status among patients with established gastric cancer [31,32,33,34,35,36,37,38,39,40]. The included studies differed substantially with respect to participant characteristics, serum vitamin D classification, laboratory assays, and adjustment strategies, highlighting the diversity of the available evidence. Circulating 25(OH)D concentrations were measured using different laboratory methods, including high-performance liquid chromatography (HPLC) [33], chemiluminescent immunoassay (CLIA) [37], radioimmunoassay (RIA) [36], enzyme-linked immunosorbent assay (ELISA) [39], and other standardised serum immunoassays [32,34,35,40].

Definitions of vitamin D status were not uniform across studies. Most investigators classified vitamin D deficiency using serum or plasma 25(OH)D concentrations below either 20 ng/mL or 50 nmol/L, whereas insufficiency was generally defined as concentrations between 20 and 30 ng/mL (50–75 nmol/L); however, several studies analysed serum vitamin D as quartiles, quintiles, continuous variables, or study-specific categories rather than predefined clinical thresholds [31,35,36,39]. Finally, although most studies investigated the association between baseline circulating 25(OH)D concentrations and gastric cancer risk, two studies evaluated prognostic outcomes among patients with established gastric cancer, focusing on overall survival, disease severity, or treatment-related outcomes rather than incident disease [38,39]. Overall, post-diagnostic case–control and cross-sectional evidence generally suggested that patients with gastric cancer had lower circulating 25(OH)D concentrations than individuals without the disease. Hospital-based case–control or cross-sectional studies from Turkey, India, the United States, and Kashmir reported significantly lower serum 25(OH)D levels or a higher prevalence of vitamin D deficiency or insufficiency among gastric cancer patients compared with control populations, despite differences in study design, sample size, laboratory methods, and vitamin D classification criteria [33,35,37,40].

Similarly, secondary biomarker analyses within a large Korean hospital-based study demonstrated significantly lower plasma 25(OH)D and 1,25(OH)_2_D concentrations among gastric cancer patients, despite no significant association between dietary vitamin D intake and gastric cancer risk [34]. In addition, a population-based cross-sectional analysis from the Korean National Health and Nutrition Examination Survey reported that higher serum 25(OH)D concentrations were independently associated with lower odds of gastric cancer prevalence after multivariable adjustment, further supporting an inverse association between circulating vitamin D status and prevalent gastric cancer [36].

In contrast, prospective investigations evaluating pre-diagnostic circulating vitamin D concentrations did not consistently demonstrate an association with subsequent gastric cancer risk. The pooled analysis by Abnet et al. and the prospective Chinese case–cohort study by Chen et al. found no significant overall association between baseline circulating 25(OH)D concentrations and gastric cancer risk, although subgroup findings suggested that associations may vary according to tumour subsite or population characteristics [31,32]. These discrepancies may reflect differences in study design, timing of vitamin D assessment, population characteristics, cancer subsite, and the distinction between pre-diagnostic vitamin D status and measurements obtained after cancer diagnosis.

Two studies specifically investigated the prognostic significance of pretreatment circulating 25(OH)D concentrations in patients with established gastric cancer, yielding conflicting findings. Ren et al. retrospectively evaluated 197 patients with gastric cancer and reported that vitamin D deficiency was common, affecting nearly 60% of the study population. Lower pretreatment serum 25(OH)D concentrations were significantly associated with more advanced clinical stage and lymph node metastasis. Patients with serum vitamin D concentrations ≥50 nmol/L had significantly better overall survival than those with deficient levels, and vitamin D status remained an independent prognostic factor after multivariable adjustment, suggesting a potential association between circulating vitamin D status, disease progression, and long-term survival [39].

In contrast, Obermannova et al. analysed pretreatment plasma 25(OH)D concentrations in 630 patients with advanced gastric or gastroesophageal junction cancer enrolled in the multicentre phase III EXPAND trial. Although severe hypovitaminosis D was highly prevalent, baseline 25(OH)D concentrations were not associated with overall survival or treatment efficacy. No interaction was observed between circulating vitamin D levels and the clinical benefit of cetuximab-containing chemotherapy. The authors concluded that, despite the high prevalence of vitamin D deficiency in advanced gastric cancer, baseline vitamin D status did not appear to influence prognosis or treatment response in this cohort [38].

The available prognostic evidence remains inconclusive. While one retrospective cohort identified pretreatment vitamin D status as an independent predictor of survival, the larger retrospective biomarker analysis embedded within a randomised clinical trial failed to confirm a prognostic effect. Differences in disease stage, patient characteristics, treatment setting, and study design may partly explain these discrepant findings.

#### 3.3.2. Meta-Analysis

**Figure 2:**
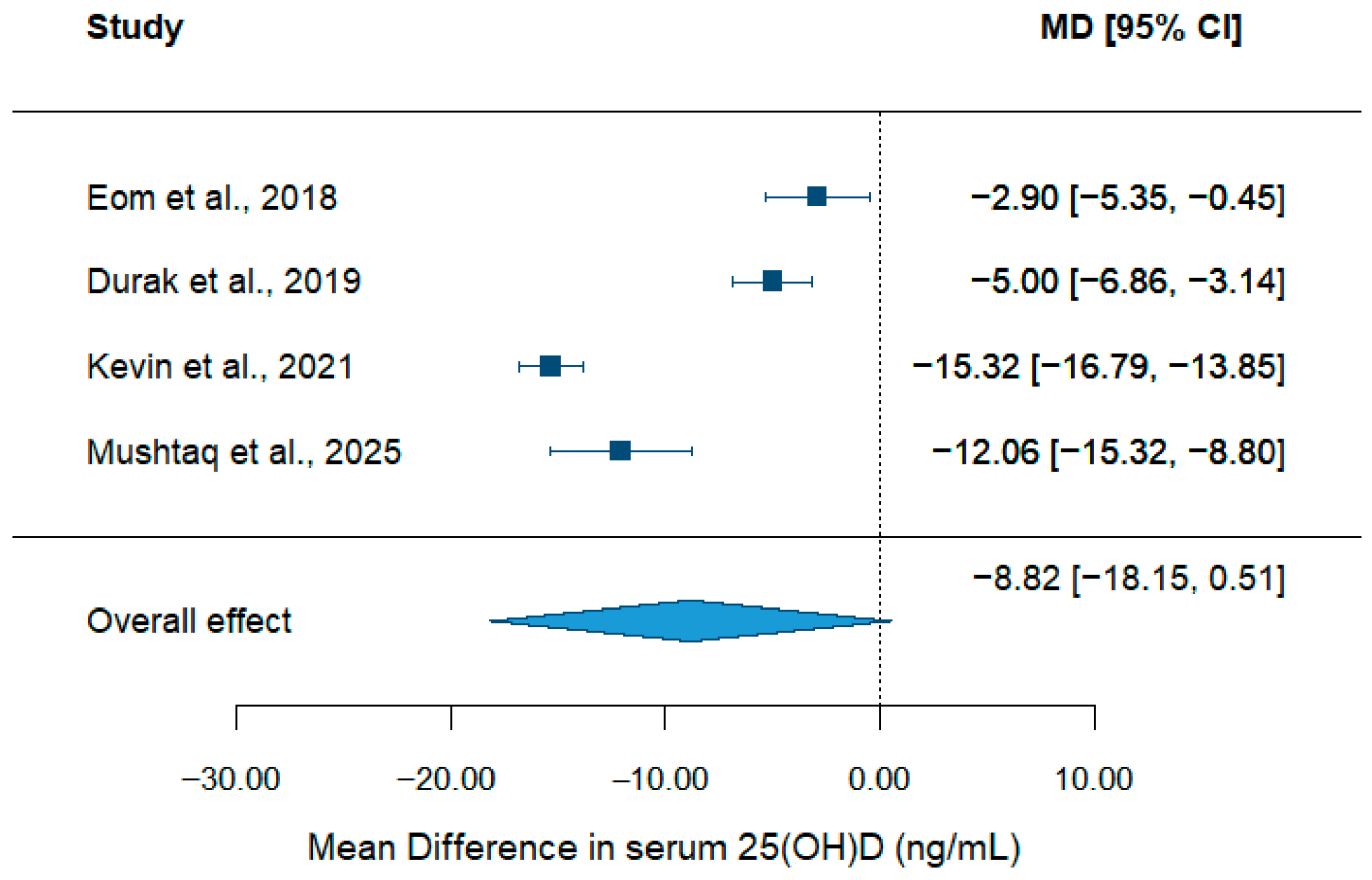
Forest plot comparing circulating serum 25(OH)D concentrations between patients with gastric cancer and controls [33,34,35,37].

Four studies comprising 302 patients with gastric cancer and 329 controls were included in the quantitative synthesis evaluating circulating serum 25(OH)D concentrations [33,34,35,37]. All studies reported serum vitamin D levels in ng/mL and compared patients with histologically confirmed gastric cancer with healthy control subjects, allowing direct pooling of mean differences using a random-effects model (Figure 2). The pooled analysis showed a lower mean circulating serum 25(OH)D concentration among patients with gastric cancer compared with control participants, with a pooled mean difference (MD) of −8.82 ng/mL (95% CI −18.15 to 0.51; *p* = 0.057). Thus, although the pooled estimate was approximately 8.8 ng/mL lower in patients with gastric cancer, the 95% confidence interval included the null value.

Substantial between-study heterogeneity was observed (Cochran’s Q = 112.14, *p* < 0.001; I^2^ = 96.68%; τ^2^ = 33.24). Nevertheless, all four studies showed the same direction of effect, with lower circulating serum vitamin D concentrations among patients with gastric cancer than among control participants. Therefore, the high heterogeneity primarily reflected differences in the magnitude of the observed effect rather than differences in its direction. The individual mean differences ranged from −2.90 ng/mL in the Korean study [34] to −15.32 ng/mL in the Indian study [35]. The 95% prediction interval ranged from −29.41 to 11.76 ng/mL, indicating substantial uncertainty regarding the magnitude of the association in a comparable future population. The inclusion of both negative and positive values within the prediction interval is consistent with the very high heterogeneity between studies and suggests that the magnitude, and potentially the direction, of the association may vary across populations and clinical settings.

To assess the robustness of the pooled estimate, a leave-one-out sensitivity analysis was performed by sequentially excluding each study. The direction of the pooled effect remained negative in all analyses, with pooled estimates ranging from −6.54 to −10.79 ng/mL. Exclusion of Kevin et al. [35] resulted in the greatest reduction in heterogeneity, with I^2^ decreasing from 96.68% to 92.78%; however, substantial heterogeneity persisted. Exclusion of Mushtaq et al. [37] yielded a pooled MD of −7.78 ng/mL (95% CI −24.33 to 8.76; *p* = 0.180), while exclusion of the other individual studies likewise resulted in confidence intervals that included the null value. These findings indicate that no single study reversed the direction of the pooled association, although the statistical uncertainty remained substantial. Taken together, the meta-analysis showed a consistent direction toward lower circulating serum 25(OH)D concentrations among patients with gastric cancer, but the pooled association did not reach conventional statistical significance and was characterised by very high between-study heterogeneity. Therefore, the magnitude of the observed association should be interpreted cautiously. The quantitative data extracted from the studies included in the meta-analysis of circulating serum 25(OH)D concentrations are presented in Table 5.

### 3.4. Dietary Vitamin D Intake and Gastric Cancer

#### 3.4.1. Narrative Synthesis

Dietary vitamin D intake or vitamin D-related dietary quality was evaluated in eight observational studies, including seven case–control studies and one prospective cohort/post hoc analysis of a randomised gastric cancer screening trial conducted in Korea, Jordan, China, Vietnam, Italy, Japan, and Iran [34,41,42,43,44,45,46,47]. Together, these studies included more than 9000 participants and reflected substantial geographical and temporal diversity in dietary assessment and gastric cancer epidemiology. Dietary vitamin D intake was predominantly assessed using validated Food Frequency Questionnaires (FFQs), although the specific instruments differed across studies. Semi-quantitative FFQs were used in the Vietnamese and Chinese studies, whereas validated interviewer-administered or locally adapted FFQs were employed in the Korean, Italian, Jordanian, and Iranian investigations [34,41,42,43,44,46]. Takasu et al. estimated habitual nutrient intake using a self-administered FFQ within a prospective screening cohort, while Vahid et al. evaluated vitamin D through the Index of Nutritional Quality (INQ), a nutrient-density measure derived from FFQ-based dietary intake rather than absolute vitamin D consumption [45,47].

The included studies also differed considerably with respect to exposure categorization and statistical adjustment. Dietary vitamin D intake was analysed using tertiles, quartiles, quintiles, high-versus-low intake categories, or nutrient quality indices, precluding direct comparison of absolute intake levels across studies. Multivariable models generally adjusted for established gastric cancer risk factors, including age, sex, body mass index, smoking status, alcohol consumption, total energy intake, socioeconomic variables, and *Helicobacter pylori* infection where available, although the specific covariates varied between studies. Overall, the findings regarding dietary vitamin D intake and gastric cancer risk were more heterogeneous than those observed for circulating serum 25(OH)D concentrations. Overall, findings regarding dietary vitamin D intake and gastric cancer risk were mixed, with some studies reporting inverse associations, others reporting null associations, and two studies reporting positive associations.

Several case–control studies consistently reported an inverse association between dietary vitamin D intake and gastric cancer risk. Nguyen et al., in the largest dietary study included, demonstrated a progressive reduction in gastric cancer risk across increasing quintiles of vitamin D intake, with participants in the highest intake category exhibiting a significantly lower risk than those in the lowest quintile (adjusted OR 0.68, 95% CI 0.53–0.86) [43]. Similarly, Allehdan et al. observed approximately 53% lower odds of gastric cancer among individuals in the highest tertile of dietary vitamin D intake compared with the lowest tertile (adjusted OR 0.47, 95% CI 0.23–0.95) [41], while Chen et al. reported a 33% reduction in gastric cancer risk associated with higher dietary vitamin D intake (adjusted OR 0.67, 95% CI 0.48–0.92) [42]. Rather than evaluating absolute vitamin D intake, Vahid et al. assessed the vitamin D Index of Nutritional Quality and found that higher vitamin D nutritional quality was independently associated with lower gastric cancer risk (adjusted OR 0.14, 95% CI 0.02–0.84), suggesting that overall nutrient density and dietary quality may also be relevant [47].

In contrast, two studies failed to identify a statistically significant association between dietary vitamin D intake and gastric cancer risk: Pelucchi et al. reported no significant relationship between vitamin D intake and gastric cancer after multivariable adjustment (OR 1.33, 95% CI 0.80–2.21), despite observing significant associations for several other micronutrients [44]. Likewise, Eom et al. found no independent association between dietary vitamin D intake and gastric cancer susceptibility, although dietary vitamin D intake showed a significant positive correlation with circulating 25(OH)D concentrations [34]. Furthermore, no evidence of interaction between dietary vitamin D intake and VDR or TXNIP polymorphisms was observed, suggesting that dietary vitamin D alone did not substantially influence gastric cancer risk within this Korean population.

Another two studies reported findings in the opposite direction: Toorang et al. observed a significantly increased risk of gastric cancer among individuals with higher dietary vitamin D intake (adjusted OR 1.59, 95% CI 1.07–2.36), while Takasu et al., in the only prospective cohort included in this subgroup, also identified higher vitamin D intake as an independent predictor of incident gastric cancer during six years of follow-up (adjusted HR 2.75, 95% CI 1.11–6.79). Both authors acknowledged that residual confounding, dietary patterns, and correlations between vitamin D intake and other nutritional factors may have influenced these unexpected findings. Notably, the Japanese cohort simultaneously identified high sodium intake as a strong independent risk factor for gastric cancer, suggesting that dietary vitamin D may have reflected broader dietary habits rather than acting as an isolated causal exposure [45].

Taken together, the dietary evidence was heterogeneous and does not support a consistent direction of association across populations. Differences in dietary assessment tools, background dietary patterns, vitamin D food sources, adjustment for confounding variables, Helicobacter pylori status, and the distinction between absolute intake and nutrient-density measures may partly explain the inconsistent findings.

#### 3.4.2. Meta-Analysis

**Figure 3:**
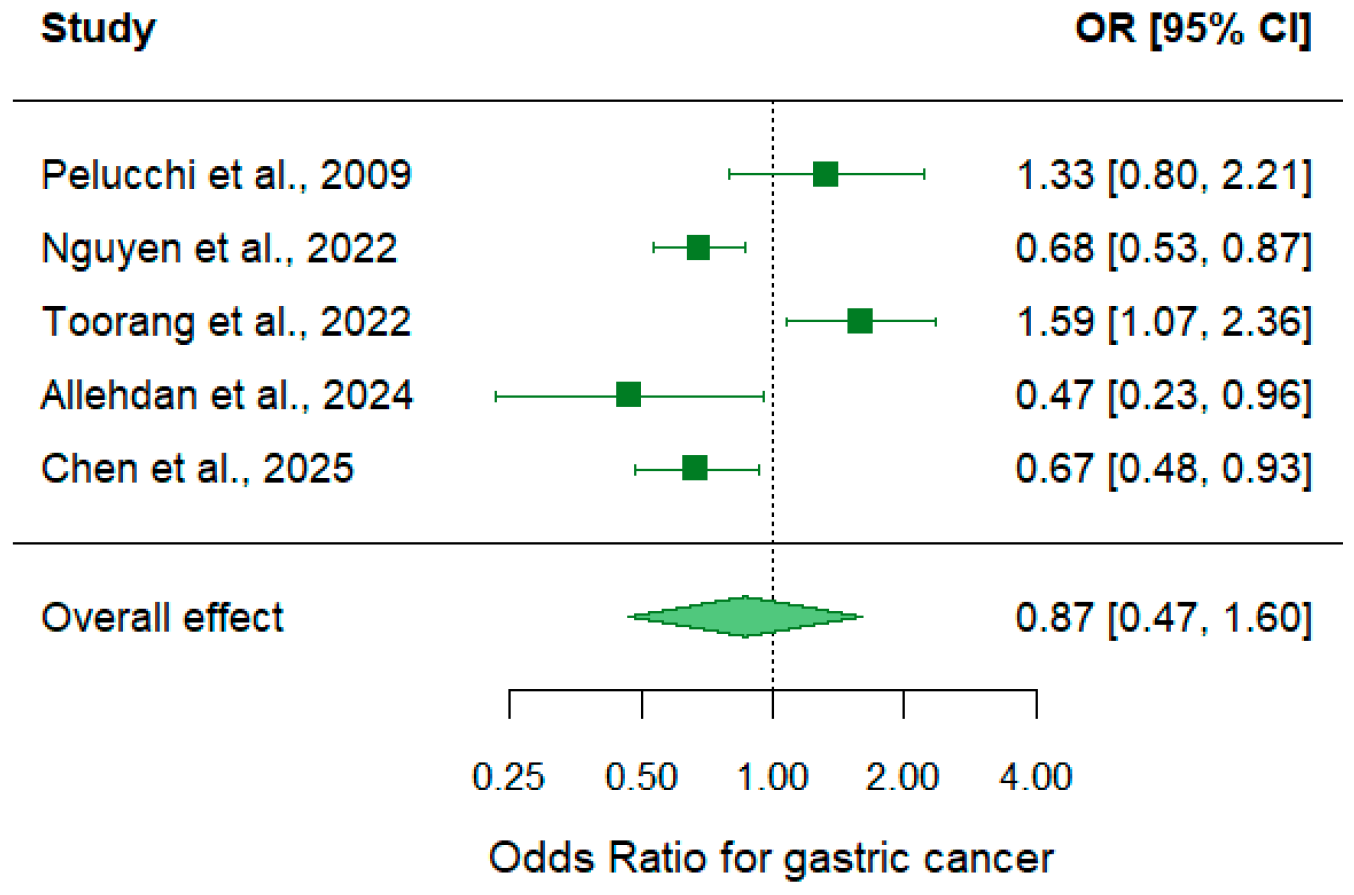
Forest plot of the association between dietary vitamin D intake and gastric cancer risk [41,42,43,44,46].

Five studies comprising a total of 1924 participants (839 gastric cancer cases and 1085 controls) were eligible for quantitative synthesis evaluating the association between dietary vitamin D intake and gastric cancer risk [41,42,43,44,46]. Because substantial clinical and methodological heterogeneity was anticipated across studies, a random-effects model was applied. The pooled analysis showed no statistically significant association between dietary vitamin D intake and gastric cancer risk, with a pooled odds ratio (OR) of 0.87 (95% CI 0.47–1.60; *p* = 0.556) (Figure 3). Although the pooled estimate was below unity, suggesting a possible trend towards a lower risk of gastric cancer with higher dietary vitamin D intake, the confidence interval included the null value, and the association did not reach conventional statistical significance. Substantial between-study heterogeneity was observed (I^2^ = 82.83%; Cochran’s Q = 20.39, *p* < 0.001; τ^2^ = 0.183), indicating considerable variability in the magnitude of the observed associations across studies. This heterogeneity may reflect differences in study populations, dietary assessment instruments, categorization of vitamin D intake, adjustment for potential confounding variables, geographical differences in dietary habits, sunlight exposure, and Helicobacter pylori prevalence.

The individual study estimates varied in both magnitude and direction. Three studies reported inverse associations between higher dietary vitamin D intake and gastric cancer risk [41,42,43], whereas one reported an association in the opposite direction [46]. Pelucchi et al. did not demonstrate a statistically significant association [44]. Thus, the observed heterogeneity was partly attributable to differences in the direction and magnitude of the study-specific estimates. The 95% prediction interval ranged from 0.23 to 3.31, indicating substantial uncertainty regarding the effect that might be observed in a future comparable population. The interval encompassed both potentially protective and potentially harmful associations, further highlighting the considerable between-study heterogeneity [41,44,46].

To assess the robustness of the pooled estimate, a leave-one-out sensitivity analysis was performed by sequentially excluding each study. The pooled association remained statistically non-significant regardless of the study omitted. Exclusion of Toorang et al. resulted in the greatest reduction in heterogeneity, with I^2^ decreasing from 82.83% to 65.43%. Following its exclusion, the pooled estimate was OR = 0.74 (95% CI 0.40–1.36; *p* = 0.214), indicating that the reduction in heterogeneity did not result in a statistically significant association. These findings suggest that no individual study changed the overall statistical conclusion, although Toorang et al. contributed substantially to the observed between-study heterogeneity [46]. The quantitative data extracted from the studies included in the meta-analysis of dietary vitamin D intake are presented in Table 6.

### 3.5. VDR Polymorphisms and Gastric Cancer Susceptibility

#### 3.5.1. Narrative Synthesis

Nine studies investigated the association between vitamin D receptor (VDR) gene polymorphisms and gastric cancer susceptibility [33,34,48,49,50,51,52,53,54]. Most studies focused on common VDR single nucleotide polymorphisms (SNPs), particularly FokI (rs2228570/rs10735810), TaqI (rs731236), ApaI (rs7975232), and BsmI (rs1544410). Additional polymorphisms were assessed in selected studies, including Cdx2 (rs11568820), rs2107301 and rs1989969 in VDR, VDBP rs7041, and TXNIP variants [33,34,51,52,54]. Overall, the available evidence comprised mainly hospital-based case–control studies from China, Turkey, Iran, India/Kashmir, and Korea, with sample sizes ranging from 161 to 1430 participants. Genotyping was performed predominantly using polymerase chain reaction-restriction fragment length polymorphism (PCR-RFLP), often followed by confirmatory DNA or Sanger sequencing, while one study used ligation detection reaction genotyping and another incorporated allele-specific PCR within a broader multi-gene susceptibility analysis [48,49,50,51,52,53,54].

Most studies evaluated the independent association between VDR polymorphisms and gastric cancer susceptibility after adjustment for potential confounding variables, including age, sex, body mass index, smoking status, alcohol consumption, family history of cancer, and, where available, clinicopathological characteristics or environmental exposures such as sunlight exposure and Helicobacter pylori infection. However, the extent of multivariable adjustment varied substantially among studies, reflecting differences in study design and available clinical information. Although several polymorphisms were investigated, FokI (rs2228570) was the only variant evaluated consistently across multiple independent cohorts, allowing quantitative synthesis. In contrast, evidence regarding TaqI, ApaI, BsmI, Cdx2 and VDBP polymorphisms remained limited to individual studies, precluding formal meta-analysis for these variants.

Among the VDR polymorphisms investigated, FokI was the most consistently evaluated variant and showed the most recurrent signal for increased gastric cancer susceptibility. Cong et al. reported that carriers of the f allele (Ff + ff) had significantly higher gastric cancer risk compared with individuals carrying the FF genotype (OR 2.73, 95% CI 1.13–4.32), and the variant genotype was also associated with poorer tumour differentiation and higher C-reactive protein concentrations [48]. Similarly, Hosseinkhani et al. observed a significant association between FokI and gastric cancer susceptibility in an Iranian Kurdish population, while Qadir et al. 2023 reported that FokI CT and TT genotypes were associated with increased gastric cancer risk in the Kashmiri population [49,52].

In contrast, Durak et al. and Parsamanesh et al. did not report statistically significant associations between FokI and gastric cancer risk, although their findings did not indicate a clearly protective direction of effect [33,50]. Yin et al. also found no overall association between FokI and gastric cardia adenocarcinoma susceptibility in a Chinese population, although subgroup and haplotype analyses suggested potential associations for other VDR loci [54]. Taken together, these findings suggest that FokI may be a relevant VDR variant for gastric cancer susceptibility, but the strength and consistency of the association vary across populations and analytic models. Evidence for TaqI was mixed. Parsamanesh et al. reported a significant association between TaqI and gastric cancer susceptibility, with increased risk for the TC genotype and the combined TC + CC genotype [50]. Shen et al. also identified VDR TaqI as one of several susceptibility genotypes associated with gastric cancer risk in a Chinese Han multi-gene model [53]. However, Durak et al., Hosseinkhani et al., and both studies of Qadir et al. did not find significant overall associations for TaqI in their respective populations [33,49,51,52]. These discrepant findings suggest that any association between TaqI and gastric cancer risk may be population-specific or dependent on the genetic model used.

Findings for BsmI and ApaI were also inconsistent. Hosseinkhani et al. found no significant association between BsmI or ApaI and gastric cancer susceptibility [49]. In contrast, Qadir et al. 2021 reported that BsmI variant genotypes were significantly associated with increased gastric cancer risk in the Kashmiri population, and that BsmI, ApaI, and TaqI variant genotypes were associated with reduced overall survival in survival analyses [51]. These findings suggest that BsmI may be relevant in some populations, but the available evidence remains limited and requires replication. Other variants were evaluated less frequently. Qadir et al. 2023 found no significant overall association for Cdx2, although specific VDR haplotypes were over-represented among gastric cancer cases [52]. Yin et al. evaluated VDR rs2107301, FokI/rs2228570, rs1989969, and Cdx2/rs11568820 in gastric cardia adenocarcinoma and found no significant overall association for the assessed SNPs. However, stratified analyses suggested increased risk associated with rs1989969 among younger individuals and alcohol drinkers, while one VDR haplotype was associated with reduced susceptibility [54]. In Eom et al., VDR and TXNIP polymorphisms did not significantly modify the association between dietary vitamin D intake and gastric cancer risk [34]. Durak et al. also evaluated VDBP rs7041 alongside VDR polymorphisms and found no independent association between these genetic variants and gastric cancer susceptibility [33].

Overall, the available genetic evidence remains heterogeneous, but FokI appears to be the most frequently investigated VDR polymorphism with the most recurrent association signal for gastric cancer susceptibility. Evidence for TaqI, BsmI, ApaI, Cdx2, VDBP, and TXNIP variants remains less consistent and is largely limited to individual studies or population-specific findings. Consequently, only studies evaluating FokI using sufficiently comparable genetic models were considered appropriate for quantitative synthesis, whereas other vitamin D-related genetic variants were retained for narrative interpretation.

#### 3.5.2. Meta-Analysis

**Figure 4:**
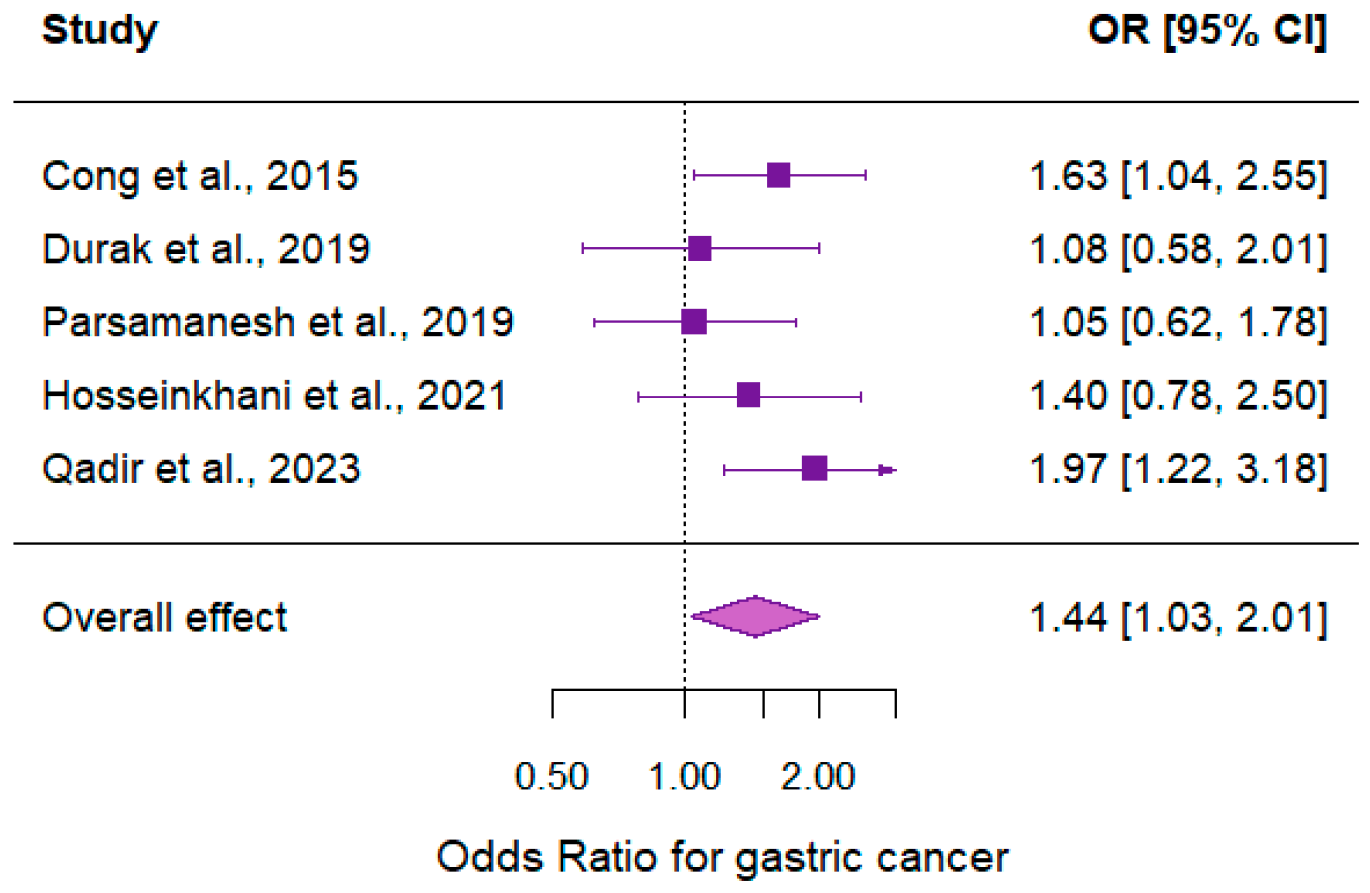
Forest plot of the association between the VDR FokI polymorphism and gastric cancer susceptibility under the dominant genetic model [33,48,49,50,52].

Five studies comprising 609 patients with gastric cancer and 693 healthy controls were included in the quantitative synthesis evaluating the association between the VDR FokI (rs2228570) polymorphism and gastric cancer susceptibility under the dominant genetic model (variant carriers versus wild-type homozygotes) [33,48,49,50,52]. Genotype frequencies were extracted directly from each study and pooled using a random-effects model (Figure 4). The pooled analysis demonstrated a statistically significant association between the VDR FokI dominant genetic model and gastric cancer susceptibility, with an odds ratio (OR) of 1.44 (95% CI 1.03–2.01; *p* = 0.040). Overall, carriers of the variant alleles had approximately 44% higher odds of gastric cancer compared with individuals carrying the CC genotype. Between-study heterogeneity was low (Cochran’s Q = 4.16, *p* = 0.385; I^2^ = 7.19%; τ^2^ = 0.0055), indicating relatively consistent effect estimates across the included studies. All five studies reported ORs above 1.0, although the magnitude and statistical precision of the individual associations varied.

Inspection of the forest plot showed that two studies reported statistically significant associations [48,52], whereas the other ones reported non-significant associations [33,49,50]. Nevertheless, all individual point estimates were above the null value, ranging from OR = 1.05 in Parsamanesh et al. [50] to OR = 1.97 in Qadir et al., [52] with no study suggesting a protective association. The 95% prediction interval ranged from 0.97 to 2.13, indicating that although the pooled analysis suggested increased gastric cancer susceptibility among variant allele carriers, the true effect in a future comparable population could potentially include a null association. Thus, the prediction interval provides a more conservative interpretation of the generalizability of the pooled genetic association despite the low observed between-study heterogeneity.

Leave-one-out sensitivity analysis demonstrated that the statistical significance of the pooled association was sensitive to the individual study composition. Exclusion of Parsamanesh et al., [50] resulted in a statistically significant pooled association (OR = 1.55, 95% CI 1.06–2.27; *p* = 0.034), whereas exclusion of each of the other four studies resulted in confidence intervals crossing the null value. The pooled estimates after individual study exclusion ranged from OR = 1.31 to OR = 1.55. These findings indicate that, despite the low between-study heterogeneity and consistent direction of effect, the statistical significance of the overall association should be interpreted with caution. The quantitative data extracted from the studies included in the meta-analysis of the VDR FokI polymorphism under the dominant genetic model are presented in Table 7.

## 4. Discussion

### 4.1. Main Findings

This systematic review integrated three complementary dimensions of vitamin D exposure—circulating 25(OH)D concentrations, dietary vitamin D intake, and VDR genetic polymorphisms—to characterise their relationship with gastric cancer. The strength and consistency of the associations differed markedly across these dimensions: the meta-analysis of the VDR FokI polymorphism showed a significant association with gastric cancer susceptibility, characterised by low between-study heterogeneity, whereas the pooled analyses of circulating 25(OH)D concentrations and dietary vitamin D intake showed non-significant associations accompanied by substantial heterogeneity.

This pattern indicates that the three exposures capture distinct, only partially overlapping biological information. Dietary intake is an indirect proxy for vitamin D status, subject to measurement error and confounded by regional dietary habits, sunlight exposure and other environmental factors, whereas circulating 25(OH)D concentrations and VDR genotype more directly reflect the vitamin D signalling pathway. The absence of a statistically significant pooled association for serum 25(OH)D nonetheless indicates that the current evidence is insufficient to establish a definitive relationship between circulating vitamin D status and gastric cancer risk. Rather than functioning as an isolated nutritional exposure, vitamin D may be more informative as one component of a broader signalling network involving receptor activity and host susceptibility; accordingly, circulating 25(OH)D status should be regarded as complementary to, rather than a substitute for, dietary vitamin D assessment when investigating gastric carcinogenesis [10,55].

### 4.2. Biological Plausibility

#### 4.2.1. Experimental and Mechanistic Evidence

**Cell proliferation, apoptosis and cell-cycle control:** In gastric cancer cell lines, calcitriol (1,25-dihydroxyvitamin D3) inhibits proliferation by inducing cell-cycle arrest and promoting apoptosis, effects mediated through enhanced PTEN activity and suppression of PI3K/AKT signalling [56,57]. These antiproliferative effects are accompanied by increased sensitivity to cisplatin, suggesting a possible chemosensitizing role [58,59].

**Epigenetic and long non-coding RNA-mediated regulation:** Vitamin D modulates BMP3 promoter methylation, restoring tumour-suppressor gene expression and potentially delaying malignant progression [60,61]. VDR-associated long non-coding RNAs, including SNHG6 and SNHG16, have similarly been implicated in gastric cancer development through dysregulation of vitamin D signalling and downstream oncogenic pathways [62,63,64], indicating that disruption of VDR activity may impair antitumour mechanisms at multiple regulatory levels [65,66].

**Invasion, metastasis and angiogenesis:** VDR activation suppresses Wnt/β-catenin signalling, limiting epithelial–mesenchymal transition and reducing gastric cancer cell migration and invasion, and may inhibit angiogenesis through downregulation of HIF-1α and VEGF [10,14,67,68]. By preventing nuclear accumulation of β-catenin, vitamin D reduces transcription of oncogenic targets such as c-Myc and restores epithelial integrity via increased E-cadherin expression [69,70].

**Figure 5:**
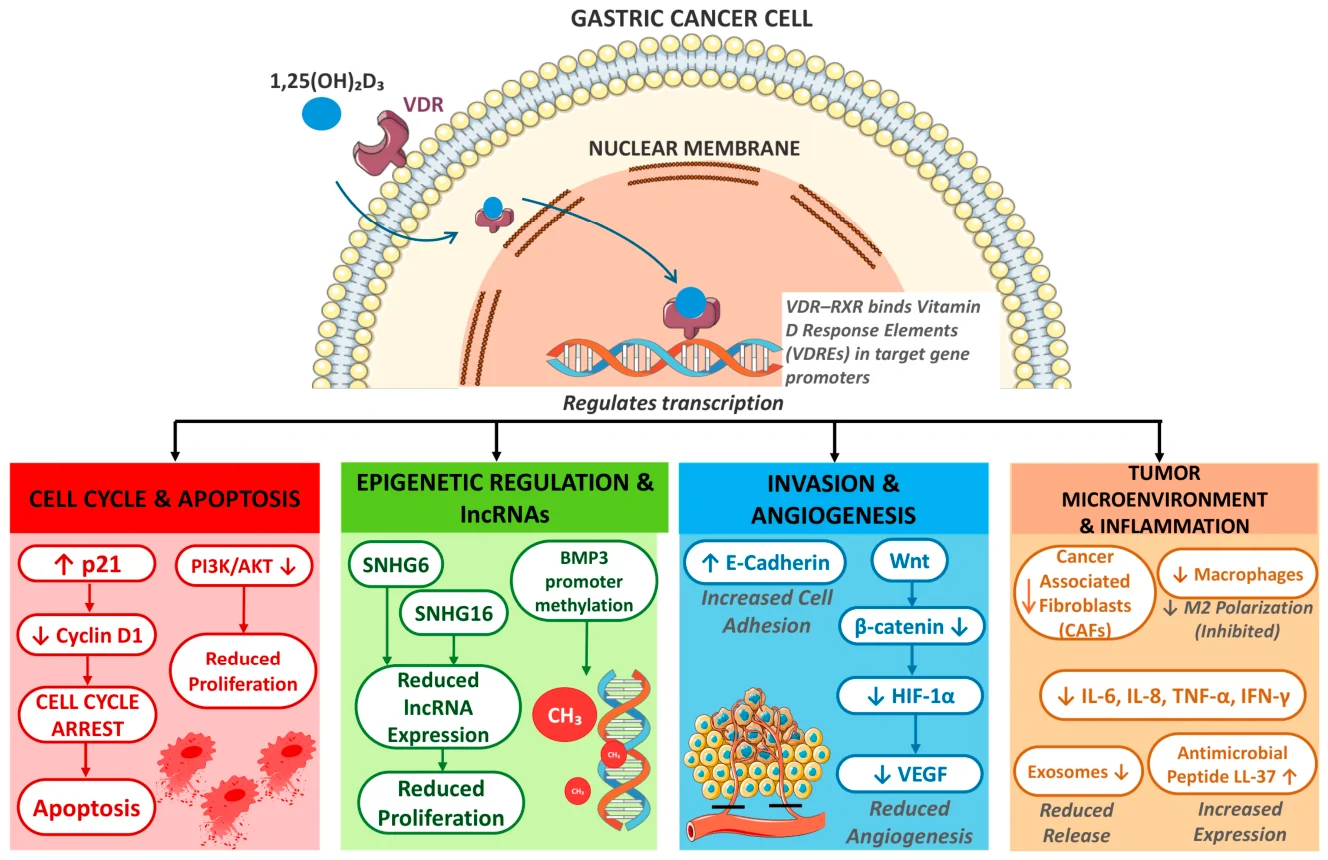
Overview of vitamin D receptor (VDR)-mediated signalling pathways implicated in gastric carcinogenesis. Following ligand binding, the vitamin D–VDR complex translocates to the nucleus, where it binds Vitamin D Response Elements (VDREs) to regulate transcription of target genes across four interconnected mechanisms: cell cycle arrest and apoptosis, epigenetic regulation via long non-coding RNAs, inhibition of invasion and angiogenesis, and modulation of the tumour microenvironment and inflammation. Arrows indicate the direction of the signaling pathways; ↑ and ↓ indicate increased and decreased expression or activity, respectively.

**Tumour microenvironment and immune-mediated mechanisms:** Vitamin D may modulate the tumour microenvironment by reducing cancer-associated fibroblast activity and promoting tumour-suppressive exosome signalling [71], and by enhancing protective autophagy through the ATG13–Beclin1 pathway, while deficiency has been associated with impaired cellular stress responses and greater tumour progression in experimental models [72]. Vitamin D also contributes to gastric epithelial barrier integrity and regulates innate and adaptive immune responses, including production of the antimicrobial peptide LL-37; combined vitamin D and probiotic supplementation has been reported to reduce circulating IL-6, IL-8, TNF-α and IFN-γ concentrations, suggesting a possible adjunctive role in limiting Helicobacter pylori-associated gastric mucosal injury [73,74,75,76]. These vitamin D–mediated mechanisms and their potential roles in gastric carcinogenesis are summarized in Figure 5.

#### 4.2.2. Tissue-Based and Clinical Correlative Evidence

Consistent with these mechanistic data, VDR expression is reduced in gastric adenocarcinoma tissue compared with adjacent normal mucosa, and lower expression correlates with more aggressive disease and poorer clinical outcomes [77]. A comparable inverse relationship between tumour VDR expression and prognosis has been reported in paediatric solid tumours other than gastric cancer, indicating that this mechanism may not be gastric cancer-specific [78]. These tissue-based observations align with the present genetic meta-analysis, in which the FokI polymorphism—which affects VDR protein structure and function—was significantly associated with gastric cancer susceptibility, reinforcing the possibility that receptor functionality contributes to gastric cancer risk independently of circulating 25(OH)D concentrations [20].

Collectively, this evidence provides biological plausibility for a role of vitamin D signalling in gastric carcinogenesis. Much of it derives from in vitro gastric cancer models or from tumour types other than gastric cancer and cannot by itself establish causality; combined with the heterogeneous epidemiological findings above, it should be regarded as hypothesis-generating rather than confirmatory.

### 4.3. Comparison with Previous Systematic Reviews, Meta-Analyses, and Broader Literature

Recent methodological discussions have highlighted the limitations of traditional randomised controlled trials for evaluating threshold-dependent micronutrients such as vitamin D, underscoring the continued value of well-conducted observational evidence and meta-analyses in this field [79].

**Comparison of serum 25**(**OH**)**D findings:** Our findings are broadly consistent with previous meta-analyses reporting lower circulating 25(OH)D concentrations among patients with gastric cancer [19,80]. Although the pooled association in the present review did not reach statistical significance, most post-diagnostic case–control and cross-sectional studies reported lower circulating 25(OH)D concentrations among patients with gastric cancer, whereas prospective investigations of pre-diagnostic 25(OH)D concentrations showed less consistent associations. The overall pattern is also consistent with evidence suggesting that vitamin D deficiency and altered calcium homeostasis may contribute to gastric carcinogenesis [81]. Compared with the earlier review by Khayyatzadeh et al., [17] which reported insufficient evidence for a clear association, the larger body of observational data currently available appears to support a more consistent, although still inconclusive, relationship between vitamin D status and gastric cancer. Evidence from intervention studies should be interpreted cautiously, as trial design, dosing strategy, and baseline vitamin D status may substantially influence outcomes; daily supplementation appears to produce more favourable cancer-related outcomes than intermittent bolus administration [82]. Vitamin D deficiency also remains highly prevalent among patients following gastrectomy [83] and among paediatric oncology patients [84], and it has been linked to poorer survival outcomes in these groups [85], reinforcing the case for routine vitamin D status monitoring across the gastric cancer care pathway.

**Comparison of dietary vitamin D intake and UVB exposure:** In agreement with our findings, the Stomach Cancer Pooling (StoP) Project reported no significant association between dietary vitamin D intake and gastric cancer risk [86]. The absence of a significant dietary association may partly reflect methodological limitations inherent to nutritional epidemiology, including recall bias, measurement error in food-frequency questionnaires, and changes in dietary habits over time; this interpretation is supported by our sensitivity analysis, in which exclusion of the study by Toorang et al. [46] substantially reduced heterogeneity and shifted the pooled estimate toward a protective association, although statistical significance remained absent. In contrast, evidence relating to solar ultraviolet-B (UVB) radiation, the major source of endogenous vitamin D synthesis, appears more consistent: we corroborate the meta-analysis by Chen et al., which reported that higher ambient UVB exposure was associated with a lower incidence of gastric cancer (ES = 0.86) [23], a finding further supported by environmental and ecological studies [87,88]. Together, these observations suggest that circulating status and UVB-related synthesis may capture biologically relevant exposure more accurately than dietary intake alone.

**Comparison of genetic polymorphisms** (**VDR**)**:** The most consistent quantitative findings of the present review were observed for VDR genetic variation. While previous umbrella reviews classified the evidence for VDR polymorphisms as limited or intermediate [89], and broader gastrointestinal cancer analyses reported variable associations across cancer sites, our results support a significant association between the FokI polymorphism and gastric cancer susceptibility, consistent with a recent meta-analysis identifying FokI and TaqI as potentially relevant variants in gastric carcinogenesis [20]. Unlike the serum and dietary analyses, the genetic meta-analysis demonstrated low study heterogeneity, with effect estimates in the same direction across studies, supporting the potential biological relevance of VDR-mediated signalling and suggesting that inherited variation affecting receptor function may represent a relatively stable determinant of gastric cancer susceptibility. These observations are further supported by evidence indicating that vitamin D bioavailability and downstream biological activity may be influenced by Vitamin D Binding Protein (VDBP) isoforms and related components of the vitamin D signalling pathway [90].

The available evidence suggests that serum vitamin D concentrations, dietary exposure, and VDR genetic variation should not be viewed as isolated factors, but rather as interconnected components of a broader biological system involved in immune regulation, epithelial homeostasis, and gastric carcinogenesis.

### 4.4. Childhood Vitamin D Status and Gastric Carcinogenesis: A Life-Course Hypothesis

One of the initial objectives of the present systematic review was to evaluate the relationship between vitamin D and gastric cancer across both adult and paediatric populations. However, no eligible paediatric studies fulfilled the inclusion criteria, highlighting an important gap in the current literature. This was not unexpected, given that paediatric gastric cancer is exceptionally rare and is more commonly associated with hereditary syndromes and genetic predisposition than with long-term environmental or nutritional exposures [91]. The absence of these studies should not be interpreted as evidence against a biological role of vitamin D; rather, it reflects the exceptionally long latency of gastric carcinogenesis. According to the well-established Correa cascade, gastric adenocarcinoma develops through a multistep sequence beginning with chronic active gastritis, followed by glandular atrophy, intestinal metaplasia, dysplasia and ultimately invasive carcinoma, evolving over two to four decades [68,91]. Because no eligible paediatric gastric cancer studies exist, the remainder of this section synthesises indirect evidence from paediatric oncology (predominantly non-gastric malignancies) and life-course epidemiology into a hypothesis regarding early-life vitamin D exposure and gastric carcinogenesis; it is presented explicitly as hypothesis-generating rather than as direct evidence.

Importantly, several initiating events within the Correa cascade occur during childhood. Helicobacter pylori, the strongest established risk factor for non-cardia gastric cancer, is commonly acquired during early life and may persist for decades if left untreated, inducing chronic inflammation, progressive epithelial injury and immune dysregulation that establishes the biological substrate upon which gastric cancer may eventually develop [75,92,93].

#### 4.4.1. Vitamin D Deficiency During Childhood as a Potential Early Carcinogenic Exposure

Vitamin D deficiency is highly prevalent from early childhood onward and may influence multiple biological pathways involved in carcinogenesis [84,94]. Beyond its role in skeletal development, VDR signalling regulates epithelial barrier integrity, immune function, antimicrobial peptide production, inflammatory responses, and gastrointestinal mucosal homeostasis [75,76], and alterations in VDR-mediated pathways have also been linked to paediatric tumour biology through effects on inflammation, apoptosis, cell-cycle regulation, and oncogenic signalling [95]. Prolonged hypovitaminosis D during childhood may therefore create a pro-inflammatory mucosal environment characterised by impaired epithelial repair, persistent immune activation and increased susceptibility to chronic infection [65,94,95]. Given that H. pylori infection is usually acquired during childhood, concurrent long-term vitamin D deficiency could theoretically amplify gastric mucosal inflammation from the earliest stages of life; although this hypothesis has not yet been confirmed epidemiologically, current experimental and clinical evidence supports its biological plausibility.

#### 4.4.2. Evidence from Paediatric Oncology (Non-Gastric Malignancies)

Recent systematic reviews report a high prevalence of hypovitaminosis D among children with cancer [94], while individual studies have also linked altered vitamin D status with adverse clinical and prognostic features [96,97]. This is consistent with broader paediatric data showing that vitamin D insufficiency is particularly common among younger populations [98] and has been investigated in relation to long-term health outcomes [99]. Multiple studies have identified vitamin D deficiency as one of the most common nutritional abnormalities among paediatric oncology patients, emphasising the need for standardised screening and supplementation protocols [84,100,101,102,103,104,105]. Aristizabal et al. reported that approximately two-thirds of children newly diagnosed with cancer presented with vitamin D insufficiency or deficiency before initiation of treatment; lower vitamin D concentrations were already evident at diagnosis, before chemotherapy or radiotherapy, indicating that hypovitaminosis D cannot be explained exclusively by treatment-related toxicity or nutritional deterioration [106,107]. Comparable observations have been reported in low- and middle-income countries: Mohan et al. found that 80.39% of Indian children with cancer exhibited vitamin D insufficiency despite abundant year-round sunlight exposure, a “sunlight paradox” most pronounced among older children and those with haematological malignancies, underscoring that inadequate vitamin D status is a global problem extending beyond geographical UV variation [108].

#### 4.4.3. Prognostic Significance

Beyond its high prevalence, vitamin D status may also possess prognostic significance. Juhász et al. demonstrated significantly lower pretreatment serum 25(OH)D concentrations among children with solid tumours, together with an association between vitamin D deficiency and adverse clinicopathological features and between reduced tumour VDR expression and poorer outcomes—paralleling the present genetic meta-analysis, in which VDR-affecting genetic variation (FokI) was consistently associated with gastric cancer susceptibility, and suggesting that preservation of VDR signalling, alongside adequate circulating vitamin D, is an additional determinant of cancer progression [78,109]. Kárász et al. similarly reported that lower pretreatment serum vitamin D concentrations were associated with shorter relapse-free and overall survival among children with solid tumours [110]. Kittivisuit et al. further showed that childhood cancer survivors continue to exhibit a high prevalence of vitamin D deficiency years after completing therapy, with potential consequences for bone health, metabolic status, chronic inflammation and quality of life, supporting long-term monitoring beyond active treatment [111].

#### 4.4.4. Life-Course Implications

These paediatric observations, together with the findings of the present systematic review, are compatible with a hypothesis in which long-term systemic vitamin D status, rather than dietary intake alone, may represent the biologically relevant exposure throughout carcinogenesis [65,76,112]. This aligns with life-course epidemiology, in which nutritional factors during critical developmental periods may induce long-lasting metabolic, immunological and epigenetic changes persisting into adulthood [112,113]; the 65-year follow-up of the Boyd Orr cohort, for instance, showed that childhood dietary patterns exert measurable effects on chronic disease risk decades later [114]. Taken together, current evidence is compatible with, rather than confirmatory of, a life-course model in which prolonged vitamin D deficiency interacts with chronic inflammation, genetic susceptibility, and environmental exposures across decades, potentially contributing to gastric carcinogenesis—a hypothesis that remains to be tested directly in cohorts linking paediatric vitamin D status to adult gastric cancer outcomes.

### 4.5. Strengths, Limitations and Future Directions

To our knowledge, this is among the first studies to comprehensively evaluate the relationship between vitamin D and gastric cancer by integrating three complementary dimensions of vitamin D biology. Quantitative meta-analyses were performed separately for each exposure, allowing a more precise assessment of their individual associations with gastric cancer risk, and methodological rigour was further strengthened by adherence to PRISMA guidelines, standardised risk-of-bias assessment, and sensitivity analyses.

Several limitations should be acknowledged. Most included studies were observational, precluding causal inference and leaving the findings susceptible to residual confounding. Substantial heterogeneity was observed in the serum and dietary vitamin D meta-analyses, likely reflecting differences in study populations, assessment methods, exposure categorization, and confounder adjustment, and the limited number of studies available for quantitative synthesis restricted subgroup and meta-regression analyses. The evidence base was largely derived from Asian and Middle Eastern populations, potentially limiting generalizability. Finally, no eligible paediatric studies specifically addressing gastric cancer were identified, preventing direct evaluation of early-life vitamin D exposure in gastric carcinogenesis.

Future research should prioritise large prospective cohorts with repeated assessments of circulating vitamin D concentrations across the life course, additional studies of VDR polymorphisms and gene-environment interactions, and closer attention to the interplay between vitamin D status, Helicobacter pylori infection, and host genetic susceptibility. Future studies should also directly evaluate the independent contribution of body mass index and metabolic syndrome to the vitamin D-gastric cancer relationship, as this could not be assessed from the data currently available. Randomised clinical trials employing physiologically appropriate daily supplementation regimens, and studies evaluating childhood vitamin D status as a determinant of adult gastric cancer risk, may provide valuable insights into future prevention strategies.

## 5. Conclusions

This systematic review highlights the complex relationship between vitamin D and gastric cancer. While the meta-analysis of circulating serum 25(OH)D concentrations suggested a tendency toward lower vitamin D levels among patients with gastric cancer, the pooled association did not reach statistical significance and was characterised by substantial heterogeneity. Similarly, no significant association was observed between dietary vitamin D intake and gastric cancer risk. In contrast, the VDR FokI polymorphism showed a significant association with gastric cancer susceptibility, supporting the potential importance of vitamin D signalling pathways in gastric carcinogenesis. Together with the available experimental evidence, these findings support the biological plausibility of a role for vitamin D in gastric cancer development. However, the current evidence remains predominantly observational and does not allow causal conclusions. Further high-quality prospective studies are needed to clarify the contribution of vitamin D status, genetic susceptibility, and vitamin D signalling to gastric carcinogenesis and to determine whether these pathways may have future preventive or clinical relevance.

## Figures and Tables

**Table 1:** Study characteristics.

Study	Country	Study Design	Cases/ Controls	Vitamin D Exposure	Exposure Assessment	Main Outcome	Meta-Analysis
Abnet et al., 2010 [31]	China, Finland, USA	Nested case–control within prospective cohorts	784/1066	Serum 25(OH)D	Serum 25(OH)D	No association between serum 25(OH)D and GC risk	No
Chen et al., 2007 [32]	China	Prospective case–cohort	434/1105	Serum 25(OH)D	Serum 25(OH)D	No association: cardia vs. non-cardia	Yes
Durak et al., 2019 [33]	Turkey	Case–control	77/84	Serum 25(OH)D + VDR/VDBP polymorphisms	HPLC; PCR-RFLP (VDR, VDBP)	Lower serum 25(OH)D in cases; no VDR polymorphism association	Yes (serum, FokI)
Eom et al., 2018 [34]	Korea	Hospital-based case–control	715/715 (serum subset: 72/91)	Dietary vitamin D intake + serum 25(OH)D + VDR/TXNIP polymorphisms	FFQ; 25(OH)D and 1,25(OH)_2_D; VDR/TXNIP genotyping	No dietary association; lower plasma 25(OH)D/1,25(OH)_2_D in cases; no VDR × intake interaction	Yes (serum, dietary)
Kevin et al., 2021 [35]	India	Cross-sectional	94/94	Serum 25(OH)D	Serum 25(OH)D	Lower serum 25(OH)D in cases, linked to advanced disease	Yes
Kwak et al., 2020 [36]	Korea	Population-based cross-sectional	218/32,901	Serum 25(OH)D	RIA	Higher serum 25(OH)D → lower GC prevalence	No
Mushtaq et al., 2025 [37]	India	Case–control	59/60	Serum 25(OH)D	CLIA	Lower serum 25(OH)D in GC patients vs. controls	Yes
Obermannova et al., 2019 [38]	Global/EXPAND trial sites	Prospective cohort (prognostic)	630 (GC/GEJ)	Serum 25(OH)D	CLIA	Severe hypovitaminosis D common; no association with survival or cetuximab efficacy	Prognostic only
Ren et al., 2012 [39]	China	Prospective cohort (prognostic)	197 GC	Serum 25(OH)D	Serum 25(OH)D	Low serum 25(OH)D linked to poorer prognosis/survival	Prognostic only
Vyas et al., 2016 [40]	United States	Case–control	49/49	Serum 25(OH)D	Serum 25(OH)D	Lower serum 25(OH)D in cases vs. controls	No
Allehdan et al., 2024 [41]	Jordan	Case–control	173/313	Dietary vitamin D intake	FFQ; tertiles	Higher dietary vitamin D → lower GC risk (OR 0.47, 95% CI 0.23–0.95)	Yes
Chen et al., 2025 [42]	China	Sex-matched case–control	336/336	Dietary vitamin D intake	FFQ	Higher dietary vitamin D → lower GC risk (OR 0.67, 95% CI 0.48–0.92)	Yes
Nguyen et al., 2022 [43]	Vietnam	Hospital-based case–control	1182/2995	Dietary vitamin D intake	FFQ; quintiles	Higher dietary vitamin D → lower GC risk (OR 0.68, 95% CI 0.53–0.86)	Yes
Pelucchi et al., 2009 [44]	Italy	Case–control	230/547	Dietary vitamin D intake	FFQ; quartiles	No association: dietary vitamin D vs. GC risk (OR 1.33, 95% CI 0.80–2.21)	Yes
Takasu et al., 2024 [45]	Japan	Prospective cohort (post hoc analysis of RCT)	1147 participants (25 incident GC cases)	Dietary vitamin D intake	FFQ	Higher dietary vitamin D → increased GC incidence (aHR 2.75, 95% CI 1.11–6.79)	No
Toorang et al., 2022 [46]	Iran	Hospital-based case–control	178/276	Dietary vitamin D intake	DHQ	Higher dietary vitamin D → increased GC risk (OR 1.59, 95% CI 1.07–2.36)	Yes
Vahid et al., 2018 [47]	Iran	Hospital-based case–control	82/95	Vitamin D Index of Nutritional Quality (INQ)	FFQ; INQ calculation	Higher vitamin D INQ → lower GC risk (OR 0.14, 95% CI 0.02–0.84)	No
Cong et al., 2015 [48]	China	Case–control	187/212	VDR FokI (rs10735810/rs2228570)	PCR-RFLP	FokI f-allele carriers: increased GC risk (OR 2.73, 95% CI 1.13–4.32)	Yes (FokI)
Hosseinkhani et al., 2021 [49]	Iran	Case–control	99/100	VDR FokI, ApaI, BsmI and TaqI polymorphisms	PCR-RFLP	FokI significant (OR 2.24, 95% CI 1.13–4.43); ApaI, BsmI and TaqI not significant	Yes (FokI)
Parsamanesh et al., 2019 [50]	Iran	Case–control	103/147	VDR TaqI and FokI polymorphisms	PCR-RFLP	TaqI significant (OR 2.03, 95% CI 1.19–3.47); FokI not significant	Yes (FokI)
Qadir et al., 2021 [51]	India (Kashmir)	Case–control with survival analysis	143/150	VDR BsmI, ApaI and TaqI polymorphisms	PCR-RFLP + Sanger sequencing	BsmI significant (OR 2.70, 95% CI 1.40–4.90); ApaI, TaqI not significant	No
Qadir et al., 2023 [52]	India (Kashmir)	Hospital-based case–control	143/150	VDR FokI, TaqI and Cdx2 polymorphisms	PCR-RFLP + DNA sequencing	FokI significant (OR 1.90, 95% CI 1.20–3.10); TaqI, Cdx2 not significant	Yes (FokI)
Shen et al., 2014 [53]	China	Matched case–control	564/564	VDR TaqI polymorphism (rs731236) and other susceptibility genes	PCR-RFLP + AS-PCR	TaqI independently associated with GC risk (aOR 1.89, 95% CI 1.27–2.83)	No
Yin et al., 2017 [54]	China	Hospital-based case–control	330/608	VDR polymorphisms (FokI, rs2107301, rs1989969 and rs11568820)	Ligation Detection Reaction (LDR)	No overall VDR association; rs1989969 ↑ risk in subgroups; one haplotype protective	No

Note: ↑ indicates increased risk.

**Table 2:** Summary of Newcastle–Ottawa Scale (NOS) assessment of observational studies.

Study	Selection	Comparability	Exposure/Outcome	Overall Risk of Bias
Chen et al., 2007 [32]	4/4	2/2	3/3	Low (9/9)
Abnet et al., 2010 [31]	4/4	2/2	3/3	Low (9/9)
Vyas et al., 2016 [40]	3/4	1/2	2/3	Moderate (6/9)
Eom et al., 2018 [34]	3/4	2/2	2/3	Moderate (7/9)
Durak et al., 2019 [33]	3/4	1/2	2/3	Moderate (6/9)
Mushtaq et al., 2025 [37]	3/4	1/2	2/3	Moderate (6/9)
Pelucchi et al., 2009 [44]	3/4	2/2	3/3	Low (8/9)
Vahid et al., 2018 [47]	4/4	2/2	2/3	Low (8/9)
Nguyen et al., 2022 [43]	3/4	2/2	2/3	Moderate (7/9)
Toorang et al., 2022 [46]	4/4	2/2	2/3	Low (8/9)
Allehdan et al., 2024 [41]	4/4	2/2	2/3	Low (8/9)
Takasu et al., 2024 [45]	4/4	2/2	3/3	Low (9/9)
Chen et al., 2025 [42]	4/4	2/2	2/3	Low (8/9)
Shen et al., 2014 [53]	4/4	1/2	2/3	Moderate (7/9)
Cong et al., 2015 [48]	4/4	2/2	2/3	Low (8/9)
Yin et al., 2017 [54]	4/4	2/2	2/3	Low (8/9)
Parsamanesh et al., 2019 [50]	4/4	1/2	2/3	Moderate (7/9)
Hosseinkhani et al., 2021 [49]	3/4	1/2	2/3	Moderate (6/9)
Qadir et al., 2021 [51]	4/4	2/2	2/3	Low (8/9)
Qadir et al., 2023 [52]	4/4	1/2	2/3	Moderate (7/9)

**Table 3:** Summary of AXIS assessment for the included cross-sectional studies.

Study	AXIS Assessment	Overall Risk of Bias
Kevin et al., 2021 [35]	Adequate reporting with minor methodological limitations	Moderate
Kwak et al., 2020 [36]	Adequate reporting with minor methodological limitations	Moderate

**Table 4:** Summary of QUIPS assessment of prognostic studies.

Study	Study Participation	Study Attrition	Prognostic Factor Measurement	Outcome Measurement	Study Confounding	Statistical Analysis	Overall
Ren et al., 2012 [39]	Low risk	Low risk	Low risk	Low risk	Low risk	Low risk	Low risk
Obermannova et al., 2019 [38]	Low risk	Low risk	Low risk	Low risk	Low risk	Low risk	Low risk

**Table 5:** Quantitative data extracted from studies included in the meta-analysis of circulating serum 25(OH)D concentrations.

Author	Year	Cases	Mean	SD	Controls	Mean	SD
Eom et al., [34]	2018	72	17.10	8.90	91	20.00	6.50
Durak et al., [33]	2019	77	11.00	6.00	84	16.00	6.00
Kevin et al., [35]	2021	94	13.83	5.97	94	29.15	4.13
Mushtaq et al., [37]	2025	59	20.15	9.77	60	32.21	8.30

**Table 6:** Effect estimates extracted from studies included in the meta-analysis of dietary vitamin D intake and gastric cancer risk.

Study	Year	OR	Lower 95% CI	Upper 95% CI
Pelucchi et al., [44]	2009	1.33	0.80	2.21
Nguyen et al., [43]	2022	0.68	0.53	0.86
Toorang et al., [46]	2022	1.59	1.07	2.36
Allehdan et al., [41]	2024	0.47	0.23	0.95
Chen et al., [42]	2025	0.67	0.48	0.92

**Table 7:** Genotype frequencies extracted from studies included in the meta-analysis of the VDR FokI polymorphism under the dominant genetic model.

Study	Year	SNP	Genetic Model	Cases Total	Cases Exposed	Controls Total	Controls Exposed
Cong et al., [48]	2015	FokI (rs2228570)	Ff + ff vs. FF	187	145	212	144
Durak et al., [33]	2019	FokI (rs2228570)	CT + TT vs. CC (equivalent dominant model)	77	40	84	42
Parsamanesh et al., [50]	2019	FokI (rs2228570)	CT + TT vs. CC (equivalent dominant model)	103	67	147	94
Hosseinkhani et al., [49]	2021	FokI (rs2228570)	Ff + ff vs. FF	99	67	100	60
Qadir et al., [52]	2023	FokI (rs2228570)	CT + TT vs. CC (equivalent dominant model)	143	99	150	80

## Data Availability

The data used for the meta-analyses were extracted from previously published studies cited in the manuscript. The extracted data and R code used for the statistical analyses are provided in the Supplementary Materials.

## Supplementary Materials

The following supporting information can be downloaded at: https://www.mdpi.com/article/10.3390/medsci14050562/s1, File S1: Literature search strategies and studies excluded in full-text screening; File S2: Risk of bias assessments; File S3: Data extraction and detailed characteristics of included studies; File S4: Detailed statistical analysis and R script; File S5: PRISMA 2020 Checklist.

## Abbreviations

The following abbreviations are used in this manuscript:

25(OH)D	25-hydroxyvitamin D
aHR	adjusted hazard ratio
AS-PCR	allele-specific polymerase chain reaction
BMP3	bone morphogenetic protein 3
CI	confidence interval
DHQ	Diet History Questionnaire
DNA	deoxyribonucleic acid
ES	effect size
FFQ	food frequency questionnaire
GC	gastric cancer
GEJ	Gastro-oesophageal junction
GLOBOCAN	Global Cancer Observatory
HIF-1α	hypoxia-inducible factor 1-alpha
HPLC	high-performance liquid chromatography
HR	hazard ratio
IFN-γ	interferon gamma
I^2^	I-squared statistic
IL-6	interleukin-6
IL-8	interleukin-8
INQ	Index of Nutritional Quality
KNHANES	Korean National Health and Nutrition Examination Survey
LDR	ligation detection reaction
LL-37	cathelicidin antimicrobial peptide
lncRNAs	long non-coding RNAs
MD	mean difference
NF-κB	nuclear factor kappa B
OR	odds ratio
PCR-RFLP	polymerase chain reaction-restriction fragment length polymorphism
PI3K/AKT	phosphoinositide 3-kinase/protein kinase B
PRISMA	Preferred Reporting Items for Systematic Reviews and Meta-Analyses
PTEN	phosphatase and tensin homologue
RCT	randomised controlled trial
REML	restricted maximum-likelihood
RIA	radioimmunoassay
RR	risk ratio
SD	standard deviation
SNPs	single nucleotide polymorphisms
TNF-α	tumour necrosis factor-alpha
TXNIP	thioredoxin-interacting protein
VDBP	vitamin D-binding protein
VDR	vitamin D receptor
VEGF	vascular endothelial growth factor
