# Supplementary material for: Vitamin D and Gastric Cancer: A Systematic Review and Meta-Analysis
Source: Med Sci (Basel). 2026 Sep 11;14(5):562. doi: 10.3390/medsci14050562 (PMC13608993; doi:10.3390/medsci14050562)
Supplement: Supplementary file 1 [file medsci-14-00562-s001.zip › medsci-4532650 Supplementary File S1.pdf]

Systematic Review

Vitamin D and Gastric Cancer: A Systematic Review and Meta-Analysis

Database	Search strategy	Filters applied	Records retrieved
**PubMed**	#1 ("stomach neoplasms"[MeSH Terms] OR "gastric cancer"[Title/Abstract] OR "stomach cancer"[Title/Abstract] OR "gastric carcinoma"[Title/Abstract] OR "gastric adenocarcinoma"[Title/Abstract] OR "gastric neoplasm"[Title/Abstract] OR "stomach carcinoma"[Title/Abstract])<br>#2 ("vitamin D"[MeSH Terms] OR "cholecalciferol"[MeSH Terms] OR "25-hydroxyvitamin D"[Title/Abstract] OR "25(OH)D"[Title/Abstract] OR "vitamin D receptor"[Title/Abstract] OR "VDR"[Title/Abstract] OR "VDR polymorphism"[Title/Abstract] OR "VDR gene"[Title/Abstract] OR "calcitriol"[Title/Abstract] OR "vitamin D deficiency"[Title/Abstract] OR "vitamin D supplementation"[Title/Abstract] OR "vitamin D status"[Title/Abstract] OR "vitamin D intake"[Title/Abstract] OR "FokI"[Title/Abstract] OR "TaqI"[Title/Abstract] OR "BsmI"[Title/Abstract] OR "ApaI"[Title/Abstract])<br>#1 AND #2	Humans; English; Publication years: 2007–2026	**91**
**Web of Science**	TS=(("gastric cancer" OR "stomach cancer" OR "gastric adenocarcinoma" OR "gastric carcinoma")<br>AND<br>("25-hydroxyvitamin D" OR "25(OH)D" OR "vitamin D deficiency" OR "vitamin D status" OR "vitamin D receptor" OR "VDR gene" OR "VDR polymorphism" OR FokI OR TaqI OR BsmI OR ApaI))<br><br>https://www.webofscience.com/wos/woscc/summary/850a2a5d-48b1-4305-9647-3504ff107a80-01c29f9e65/relevance/1	English; Article; Publication years: 2007–2026	**81**
**Scopus**	TITLE-ABS-KEY (("gastric cancer" OR "stomach cancer" OR "gastric adenocarcinoma" OR "gastric carcinoma") AND ("25-hydroxyvitamin D" OR "25(OH)D" OR "vitamin D deficiency" OR "vitamin D status" OR "vitamin D receptor" OR "VDR polymorphism" OR "VDR gene" OR FokI OR TaqI OR BsmI OR ApaI)) AND PUBYEAR > 2006 AND PUBYEAR < 2027 AND (LIMIT-TO (DOCTYPE, "ar")) AND (LIMIT-TO (LANGUAGE, "English"))	English; Article; Publication years: 2007–2026	**110**
**Lens.org**	Title: ("gastric cancer" OR ("stomach cancer" OR ("gastric adenocarcinoma" OR "gastric carcinoma"))) AND (Title: ("25-hydroxyvitamin D" OR (25(OH)D OR ("vitamin D deficiency" OR ("vitamin D receptor" OR "VDR polymorphism")))) OR Abstract: ("25-hydroxyvitamin D" OR (25(OH)D OR ("vitamin D deficiency" OR ("vitamin D receptor" OR "VDR polymorphism")))))	Journal Article; English; Publication years: 2007–2026	**62**

Abbreviations: TS = Topic Search (Title, Abstract and Keywords); TITLE-ABS-KEY = Title, Abstract and Keywords.

Studys excluded in full-text screening

Study	Reason for exclusion
Wen Y, Da M, Zhang Y, Peng L, Yao J, Duan Y.<br>Alterations in vitamin D signaling pathway in gastric cancer progression: a study of vitamin D receptor expression in human normal, premalignant, and malignant gastric tissue.<br><br>https://pubmed.ncbi.nlm.nih.gov/26722516/	Wrong outcome

Cousillas Castiñeiras A, Gallardo Martín E, Fernández Montes A, Carmona Campos M, Covela Rúa M, Salgado Fernández M, Pellón Augusto ML, Méndez Méndez JC, Brozos Vázquez N, Martínez Lago N. Vitamin D-regulated miRNA expression in tumoral and normal adjacent tissue of localized gastric cancer patients: the impact on survival and time to relapse. doi: 10.21037/tcr-24-2068	Wrong outcome
Fang F, Gao J, Han F, Wang HJ. Correlation of gastric cancer and effects of vitamin D receptor gene FokI polymorphism on C-reactive protein and D-dimer in Uygur nationality patients. https://doi.org/10.3760/cma.j.issn.0376-2491.2016.32.005	Full text not available in English
Yang Ying-yin Multi-gene risk analysis of susceptibility genes on primary gastric cancer https://www.lens.org/108-354-056-811-721	Full text not available in English
Zhang Y, Li Y, Wei Y, Cong L. Molecular Mechanism of Vitamin D Receptor Modulating Wnt/β-catenin Signaling Pathway in Gastric Cancer. 10.7150/jca.81034	In vitro study
Santucci C, Natale A, Pelucchi C, Bonzi R, Lunet N, Morais S, Vioque J, González-Palacios S, Aragonés N, Castaño-Vinyals G, Malekzadeh R, Pakseresht M, Negri E, Boffetta P, Camargo MC, Curado MP, Zhang ZF, Boccia S, La Vecchia C, Rossi M. Dietary vitamin D and gastric cancer risk within the stomach cancer pooling (StoP) project. 10.1007/s00394-025-03768-w	Secondary pooled analysis with overlapping study populations
