# Supplementary material for: Vitamin D and Gastric Cancer: A Systematic Review and Meta-Analysis
Source: Med Sci (Basel). 2026 Sep 11;14(5):562. doi: 10.3390/medsci14050562 (PMC13608993; doi:10.3390/medsci14050562)
Supplement: Supplementary file 1 [file medsci-14-00562-s001.zip › medsci-4532650 supplementary File S2.pdf]

Systematic Review

Vitamin D and Gastric Cancer: A Systematic Review and Meta-Analysis

Contents – NOS and AXIS

Contents – NOS and AXIS 1
1.NEWCASTLE - OTTAWA QUALITY ASSESSMENT SCALE CASE CONTROL STUDIES – Allehdan et al., 2024 2
2.NEWCASTLE - OTTAWA QUALITY ASSESSMENT SCALE CASE CONTROL STUDIES – Durak et al., 2019 1
3. NEWCASTLE - OTTAWA QUALITY ASSESSMENT SCALE CASE CONTROL STUDIES – Chen et al., 2025 2
4.NEWCASTLE - OTTAWA QUALITY ASSESSMENT SCALE CASE CONTROL STUDIES – Cong et al., 2015 3
5.NEWCASTLE - OTTAWA QUALITY ASSESSMENT SCALE CASE CONTROL STUDIES – Hosseinkhani et al., 2021 4
6.NEWCASTLE - OTTAWA QUALITY ASSESSMENT SCALE CASE CONTROL STUDIES – Eom et al., 2018 5
7.NEWCASTLE - OTTAWA QUALITY ASSESSMENT SCALE CASE CONTROL STUDIES – Nguyen et al., 2022 6
8.NEWCASTLE - OTTAWA QUALITY ASSESSMENT SCALE CASE CONTROL STUDIES – Parsamanesh et al., 2019 8
9.NEWCASTLE - OTTAWA QUALITY ASSESSMENT SCALE CASE CONTROL STUDIES – Mushtaq et al., 2025 9
10.NEWCASTLE - OTTAWA QUALITY ASSESSMENT SCALE CASE CONTROL STUDIES – Pelucchi et al., 2009 10
11.NEWCASTLE - OTTAWA QUALITY ASSESSMENT SCALE CASE CONTROL STUDIES – Qadir et al., 2021 11
12.NEWCASTLE - OTTAWA QUALITY ASSESSMENT SCALE CASE CONTROL STUDIES – Qadir et al., 2023 12
13.NEWCASTLE - OTTAWA QUALITY ASSESSMENT SCALE CASE CONTROL STUDIES – Toorang et al., 2022 14
14.NEWCASTLE - OTTAWA QUALITY ASSESSMENT SCALE CASE CONTROL STUDIES – Shen et al., 2014 15
15. NEWCASTLE - OTTAWA QUALITY ASSESSMENT SCALE CASE CONTROL STUDIES – Vyas et al., 2016 16
16.NEWCASTLE - OTTAWA QUALITY ASSESSMENT SCALE CASE CONTROL STUDIES – Vahid et al., 2018 17
17.NEWCASTLE - OTTAWA QUALITY ASSESSMENT SCALE CASE CONTROL STUDIES – Yin., et al 2017 18
18. NEWCASTLE - OTTAWA QUALITY ASSESSMENT SCALE COHORT STUDIES – Chen et al., 2007 19
19.NEWCASTLE - OTTAWA QUALITY ASSESSMENT SCALE COHORT STUDIES – Abnet et al., 2010 21
20.NEWCASTLE - OTTAWA QUALITY ASSESSMENT SCALE COHORT STUDIES – Takasu et al., 2024 22
Appraisal tool for Cross-Sectional Studies (AXIS) – Kevin et al., 2021 24
Appraisal tool for Cross-Sectional Studies (AXIS) – Kwak et al., 2020 25

For clarity, the corresponding responses to each AXIS checklist item are highlighted in green, whereas all information added to complete previously blank fields is presented in bold.

1. NEWCASTLE - OTTAWA QUALITY ASSESSMENT SCALE CASE CONTROL STUDIES – Allehdan et al., 2024

Note: A study can be awarded a maximum of one star for each numbered item within the Selection and Exposure categories. A maximum of two stars can be given for Comparability.

Selection

1) Is the case definition adequate?
   a) yes, with independent validation ❋
   b) yes, eg record linkage or based on self reports
   c) no description

2) Representativeness of the cases
   a) consecutive or obviously representative series of cases ❋
   b) potential for selection biases or not stated

3) Selection of Controls
   a) community controls ❋
   b) hospital controls
   c) no description

4) Definition of Controls
   a) no history of disease **(gastric cancer)** ❋
   b) no description of source

Comparability

1) Comparability of cases and controls on the basis of the design or analysis
   a) study controls for **age and sex** (Select the most important factor.) ❋
   b) study controls for any additional factor **(BMI, smoking, family history of gastric cancer)** ❋

(This criteria could be modified to indicate specific control for a second important factor.)

Exposure

1) Ascertainment of exposure
   a) secure record (eg surgical records) ❋

b) structured interview where blind to case/control status ✵
c) interview not blinded to case/control status
d) written self report or medical record only
e) no description

2) <u>Same method of ascertainment for cases and controls</u>
a) yes ✵
b) no

3) <u>Non-Response rate</u>
a) same rate for both groups ✵
b) non respondents described
c) rate different and no designation

Total: 8/9 stars

2.NEWCASTLE - OTTAWA QUALITY ASSESSMENT SCALE CASE CONTROL STUDIES –

Durak et al., 2019

Note: A study can be awarded a maximum of one star for each numbered item within the Selection and Exposure categories. A maximum of two stars can be given for Comparability.

Selection

1) Is the case definition adequate?
   a) yes, with independent validation ❋
   b) yes, eg record linkage or based on self reports
   c) no description
2) Representativeness of the cases
   a) consecutive or obviously representative series of cases ❋
   b) potential for selection biases or not stated
3) Selection of Controls
   a) community controls ❋
   b) hospital controls
   c) no description
4) Definition of Controls
   a) no history of disease **(gastric cancer)** ❋
   b) no description of source

Comparability

1) Comparability of cases and controls on the basis of the design or analysis
   a) study controls for **age and sex** (Select the most important factor.) ❋
   b) study controls for any additional factor ❋ (This criteria could be modified to indicate specific control for a second important factor.)

Exposure

1) Ascertainment of exposure
   a) secure record **(serum 25(OH)D measured by HPLC; VDR/VDBP genotyping by PCR-RFLP)** ❋
   b) structured interview where blind to case/control status ❋
   c) interview not blinded to case/control status
   d) written self report or medical record only
   e) no description

2) Same method of ascertainment for cases and controls
   a) yes ❋
   b) no

3) Non-Response rate
   a) same rate for both groups ❋
   b) non respondents described
   c) rate different and no designation

Total 6/9 stars

3. NEWCASTLE - OTTAWA QUALITY ASSESSMENT SCALE CASE CONTROL STUDIES – Chen et al., 2025

Note: A study can be awarded a maximum of one star for each numbered item within the Selection and Exposure categories. A maximum of two stars can be given for Comparability.

Selection

1) Is the case definition adequate?
   a) yes, with independent validation ❋
   b) yes, eg record linkage or based on self reports
   c) no description

2) Representativeness of the cases
   a) consecutive or obviously representative series of cases ❋
   b) potential for selection biases or not stated

3) Selection of Controls
   a) community controls ❋
   b) hospital controls
   c) no description

4) Definition of Controls
   a) no history of disease (gastric cancer) ❋
   b) no description of source

Comparability

1) Comparability of cases and controls on the basis of the design or analysis
   a) study controls for **sex(matching) and age** (Select the most important factor.) ❋
   b) study controls for any additional factor **(Smoking)** ❋ (This criteria could be modified to indicate specific control for a second important factor.)

Exposure

1) Ascertainment of exposure

a) secure record (eg surgical records) ❋

b) structured interview where blind to case/control status ❋

c) interview not blinded to case/control status

d) written self report or medical record only

e) no description

2) Same method of ascertainment for cases and controls

a) yes ❋

b) no

3) Non-Response rate

a) same rate for both groups ❋

b) non respondents described

c) rate different and no designation

Total: 8/9 stars

4.NEWCASTLE - OTTAWA QUALITY ASSESSMENT SCALE CASE CONTROL STUDIES –

Cong et al., 2015

Note: A study can be awarded a maximum of one star for each numbered item within the Selection and Exposure categories. A maximum of two stars can be given for Comparability.

Selection

1) Is the case definition adequate?

a) yes, with independent validation ❋

b) yes, eg record linkage or based on self reports

c) no description

2) Representativeness of the cases

a) consecutive or obviously representative series of cases ❋

b) potential for selection biases or not stated

3) Selection of Controls

a) community controls ❋

b) hospital controls

c) no description

4) Definition of Controls

a) no history of disease (gastric cancer) ❋

b) no description of source

Comparability

1) Comparability of cases and controls on the basis of the design or analysis
   a) study controls for **age and sex** (Select the most important factor.) ❋
   b) study controls for any additional factor **(Smoking, family history of tumors, BMI, alcohol consumption)** ❋ (This criteria could be modified to indicate specific control for a second important factor.)

Exposure

1) Ascertainment of exposure
   a) secure record **(PCR-RFLP genotyping of VDR Fokl polymorphism)** ❋
   b) structured interview where blind to case/control status ❋
   c) interview not blinded to case/control status
   d) written self report or medical record only
   e) no description
2) Same method of ascertainment for cases and controls
   a) yes ❋
   b) no
3) Non-Response rate
   a) same rate for both groups ❋
   b) non respondents described
   c) rate different and no designation

Total: 8/9 stars

5.NEWCASTLE - OTTAWA QUALITY ASSESSMENT SCALE CASE CONTROL STUDIES –

Hosseinkhani et al., 2021

Note: A study can be awarded a maximum of one star for each numbered item within the Selection and Exposure categories. A maximum of two stars can be given for Comparability.

Selection

1) Is the case definition adequate?
   a) yes, with independent validation ❋
   b) yes, eg record linkage or based on self reports
   c) no description
2) Representativeness of the cases
   a) consecutive or obviously representative series of cases ❋
   b) potential for selection biases or not stated

3) Selection of Controls

a) community controls ❋

b) hospital controls

c) no description

4) Definition of Controls

a) no history of disease **(gastric cancer)** ❋

b) no description of source

Comparability

1) Comparability of cases and controls on the basis of the design or analysis

a) study controls for **sex** (Select the most important factor.) ❋

b) study controls for any additional factor ❋ (This criteria could be modified to indicate specific control for a second important factor.)

Exposure

1) Ascertainment of exposure

a) secure record (PCR-RFLP genotyping) ❋

b) structured interview where blind to case/control status ❋

c) interview not blinded to case/control status

d) written self report or medical record only

e) no description

2) Same method of ascertainment for cases and controls

a) yes ❋

b) no

3) Non-Response rate

a) same rate for both groups ❋

b) non respondents described

c) rate different and no designation

Total 6/9 stars

6.NEWCASTLE - OTTAWA QUALITY ASSESSMENT SCALE CASE CONTROL STUDIES –

Eom et al., 2018

Note: A study can be awarded a maximum of one star for each numbered item within the Selection and Exposure categories. A maximum of two stars can be given for Comparability.

Selection

1) Is the case definition adequate?

a) yes, with independent validation ❋
b) yes, eg record linkage or based on self reports
c) no description

2) Representativeness of the cases
a) consecutive or obviously representative series of cases ❋
b) potential for selection biases or not stated

3) Selection of Controls
a) community controls ❋
b) hospital controls
c) no description

4) Definition of Controls
a) no history of disease **(gastric cancer)** ❋
b) no description of source

Comparability

1) Comparability of cases and controls on the basis of the design or analysis
a) study controls for **age and sex** (Select the most important factor.) ❋
b) study controls for any additional factor **(Smoking, alcohol intake, *Helicobacter pylori infection*)**❋ (This criteria could be modified to indicate specific control for a second important factor.)

Exposure

1) Ascertainment of exposure
a) secure record (**FFQs, plasma vitamin D measured by ELISA, genotyping using GoldenGate assay**) ❋
b) structured interview where blind to case/control status ❋
c) interview not blinded to case/control status
d) written self report or medical record only
e) no description

2) Same method of ascertainment for cases and controls
a) yes ❋
b) no

3) Non-Response rate
a) same rate for both groups ❋
b) non respondents described
c) rate different and no designation

Total: 7/9

7.NEWCASTLE - OTTAWA QUALITY ASSESSMENT SCALE CASE CONTROL STUDIES –

Nguyen et al., 2022

Note: A study can be awarded a maximum of one star for each numbered item within the Selection and Exposure categories. A maximum of two stars can be given for Comparability.

Selection

1) Is the case definition adequate?
   a) yes, with independent validation ❋
   b) yes, eg record linkage or based on self reports
   c) no description
2) Representativeness of the cases
   a) consecutive or obviously representative series of cases ❋
   b) potential for selection biases or not stated
3) Selection of Controls
   a) community controls ❋
   b) hospital controls
   c) no description
4) Definition of Controls
   a) no history of disease **(gastric cancer)** ❋
   b) no description of source

Comparability

1) Comparability of cases and controls on the basis of the design or analysis
   a) study controls for **age and sex** (Select the most important factor.) ❋
   b) study controls for any additional factor **(BMI, smoking, alcohol intake, family history, H. pylori infection)** ❋ (This criteria could be modified to indicate specific control for a second important factor.)

Exposure

1) Ascertainment of exposure
   a) secure record (eg surgical records) ❋
   b) structured interview where blind to case/control status ❋
   c) interview not blinded to case/control status
   d) written self report or medical record only
   e) no description
2) Same method of ascertainment for cases and controls
   a) yes ❋
   b) no

3) Non-Response rate
   a) same rate for both groups ❋
   b) non respondents described
   c) rate different and no designation

Total: 7/9 stars

8.NEWCASTLE - OTTAWA QUALITY ASSESSMENT SCALE CASE CONTROL STUDIES – Parsamanesh et al., 2019

Note: A study can be awarded a maximum of one star for each numbered item within the Selection and Exposure categories. A maximum of two stars can be given for Comparability.

Selection

1) Is the case definition adequate?
   a) yes, with independent validation ❋
   b) yes, eg record linkage or based on self reports
   c) no description

2) Representativeness of the cases
   a) consecutive or obviously representative series of cases ❋
   b) potential for selection biases or not stated

3) Selection of Controls
   a) community controls ❋
   b) hospital controls
   c) no description

4) Definition of Controls
   a) no history of disease (gastric cancer) ❋
   b) no description of source

Comparability

1) Comparability of cases and controls on the basis of the design or analysis
   a) study controls for **age and sex** (Select the most important factor.) ❋
   b) study controls for any additional factor ❋ (This criteria could be modified to indicate specific control for a second important factor.)

Exposure

1) Ascertainment of exposure

a) secure record **(VDR polymorphisms determined by PCR-RFLP)** ❋
b) structured interview where blind to case/control status ❋
c) interview not blinded to case/control status
d) written self report or medical record only
e) no description

2) Same method of ascertainment for cases and controls
a) yes ❋
b) no

3) Non-Response rate
a) same rate for both groups ❋
b) non respondents described
c) rate different and no designation

Total 7/9 stars

9.NEWCASTLE - OTTAWA QUALITY ASSESSMENT SCALE CASE CONTROL STUDIES – Mushtaq et al., 2025

Note: A study can be awarded a maximum of one star for each numbered item within the Selection and Exposure categories. A maximum of two stars can be given for Comparability.

Selection

1) Is the case definition adequate?
a) yes, with independent validation ❋
b) yes, eg record linkage or based on self reports
c) no description

2) Representativeness of the cases
a) consecutive or obviously representative series of cases ❋
b) potential for selection biases or not stated

3) Selection of Controls
a) community controls ❋
b) hospital controls
c) no description

4) Definition of Controls
a) no history of disease **(gastric cancer)** ❋
b) no description of source

Comparability

1) Comparability of cases and controls on the basis of the design or analysis

a) study controls for **age and sex** (Select the most important factor.) ⁕

b) study controls for any additional factor ⁕ (This criteria could be modified to indicate specific control for a second important factor.)

Exposure

1) Ascertainment of exposure

a) secure record (**Serum 25(OH)D measured using chemiluminescent immunoassay)** ⁕

b) structured interview where blind to case/control status ⁕

c) interview not blinded to case/control status

d) written self report or medical record only

e) no description

2) Same method of ascertainment for cases and controls

a) yes ⁕

b) no

3) Non-Response rate

a) same rate for both groups ⁕

b) non respondents described

c) rate different and no designation

Total: 6/9 stars

10.NEWCASTLE - OTTAWA QUALITY ASSESSMENT SCALE CASE CONTROL STUDIES –

Pelucchi et al., 2009

Note: A study can be awarded a maximum of one star for each numbered item within the Selection and Exposure categories. A maximum of two stars can be given for Comparability.

Selection

1) Is the case definition adequate?

a) yes, with independent validation ⁕

b) yes, eg record linkage or based on self reports

c) no description

2) Representativeness of the cases

a) consecutive or obviously representative series of cases ⁕

b) potential for selection biases or not stated

3) Selection of Controls

a) community controls ⁕

b) hospital controls

c) no description

4) Definition of Controls

a) no history of disease **(gastric cancer)** ❋

b) no description of source

Comparability

1) Comparability of cases and controls on the basis of the design or analysis

a) study controls for **age and sex** (Select the most important factor.) ❋

b) study controls for any additional factor **(BMI, smoking, family history)** ❋ (This criteria could be modified to indicate specific control for a second important factor.)

Exposure

1) Ascertainment of exposure

a) secure record (eg surgical records) ❋

b) structured interview where blind to case/control status ❋

c) interview not blinded to case/control status

d) written self report or medical record only

e) no description

2) Same method of ascertainment for cases and controls

a) yes ❋

b) no

3) Non-Response rate

a) same rate for both groups ❋

b) non respondents described

c) rate different and no designation

Total 8/9 stars

11.NEWCASTLE - OTTAWA QUALITY ASSESSMENT SCALE CASE CONTROL STUDIES – Qadir et al., 2021

Note: A study can be awarded a maximum of one star for each numbered item within the Selection and Exposure categories. A maximum of two stars can be given for Comparability.

Selection

1) Is the case definition adequate?

a) yes, with independent validation ❋

b) yes, eg record linkage or based on self reports

c) no description

2) <u>Representativeness of the cases</u>
a) consecutive or obviously representative series of cases ❋
b) potential for selection biases or not stated

3) <u>Selection of Controls</u>
a) community controls ❋
b) hospital controls
c) no description

4) <u>Definition of Controls</u>
a) no history of disease **(gastric cancer)** ❋
b) no description of source

Comparability

1) <u>Comparability of cases and controls on the basis of the design or analysis</u>
a) study controls for age and sex (Select the most important factor.) ❋
b) study controls for any additional factor **(BMI, smoking, family history)**❋ (This criteria could be modified to indicate specific control for a second important factor.)

Exposure

1) <u>Ascertainment of exposure</u>
a) secure record **(PCR-RFLP with Sanger sequencing confirmation)** ❋
b) structured interview where blind to case/control status ❋
c) interview not blinded to case/control status
d) written self report or medical record only
e) no description

2) <u>Same method of ascertainment for cases and controls</u>
a) yes ❋
b) no

3) <u>Non-Response rate</u>
a) same rate for both groups ❋
b) non respondents described
c) rate different and no designation

Total 8/9 stars

12.NEWCASTLE - OTTAWA QUALITY ASSESSMENT SCALE CASE CONTROL STUDIES –

Qadir et al., 2023

Note: A study can be awarded a maximum of one star for each numbered item within the Selection and Exposure categories. A maximum of two stars can be given for Comparability.

Selection

1) Is the case definition adequate?
   a) yes, with independent validation ❋
   b) yes, eg record linkage or based on self reports
   c) no description

2) Representativeness of the cases
   a) consecutive or obviously representative series of cases ❋
   b) potential for selection biases or not stated

3) Selection of Controls
   a) community controls ❋
   b) hospital controls
   c) no description

4) Definition of Controls
   a) no history of disease (**gastric cancer**) ❋
   b) no description of source

Comparability

1) Comparability of cases and controls on the basis of the design or analysis
   a) study controls for **age and sex** (Select the most important factor.) ❋
   b) study controls for any additional factor ❋ (This criteria could be modified to indicate specific control for a second important factor.)

Exposure

1) Ascertainment of exposure
   a) secure record (PCR-RFLP with DNA sequencing) ❋
   b) structured interview where blind to case/control status ❋
   c) interview not blinded to case/control status
   d) written self report or medical record only
   e) no description

2) Same method of ascertainment for cases and controls
   a) yes ❋
   b) no

3) Non-Response rate
   a) same rate for both groups ❋
   b) non respondents described
   c) rate different and no designation

Total 7/9 stars

13.NEWCASTLE - OTTAWA QUALITY ASSESSMENT SCALE CASE CONTROL STUDIES – Toorang et al., 2022

Note: A study can be awarded a maximum of one star for each numbered item within the Selection and Exposure categories. A maximum of two stars can be given for Comparability.

Selection

1) Is the case definition adequate?
   a) yes, with independent validation ❋
   b) yes, eg record linkage or based on self reports
   c) no description
2) Representativeness of the cases
   a) consecutive or obviously representative series of cases ❋
   b) potential for selection biases or not stated
3) Selection of Controls
   a) community controls (Healthy individuals accompanying relatives in the same hospital)❋
   b) hospital controls
   c) no description
4) Definition of Controls
   a) no history of disease (**gastric cancer**) ❋
   b) no description of source

Comparability

1) Comparability of cases and controls on the basis of the design or analysis
   a) study controls for **age and sex** (Select the most important factor.) ❋
   b) study controls for any additional factor (**Smoking, *H.pylori* infection)** ❋ (This criteria could be modified to indicate specific control for a second important factor.)

Exposure

1) Ascertainment of exposure
   a) secure record (eg surgical records) ❋
   b) structured interview where blind to case/control status ❋
   c) interview not blinded to case/control status
   d) written self report or medical record only
   e) no description
2) Same method of ascertainment for cases and controls

a) yes ❋

b) no

3) Non-Response rate

a) same rate for both groups ❋

b) non respondents described

c) rate different and no designation

Total 8/9

14.NEWCASTLE - OTTAWA QUALITY ASSESSMENT SCALE CASE CONTROL STUDIES – Shen et al., 2014

Note: A study can be awarded a maximum of one star for each numbered item within the Selection and Exposure categories. A maximum of two stars can be given for Comparability.

Selection

1) Is the case definition adequate?

a) yes, with independent validation ❋

b) yes, eg record linkage or based on self reports

c) no description

2) Representativeness of the cases

a) consecutive or obviously representative series of cases ❋

b) potential for selection biases or not stated

3) Selection of Controls

a) community controls ❋

b) hospital controls

c) no description

4) Definition of Controls

a) no history of disease (**gastric cancer**) ❋

b) no description of source

Comparability

1) Comparability of cases and controls on the basis of the design or analysis

a) study controls for **age and sex** (Select the most important factor.) ❋

b) study controls for any additional factor ❋ (This criteria could be modified to indicate specific control for a second important factor.)

Exposure

1) Ascertainment of exposure

a) secure record (PCR-RFLP genotyping) ❋

b) structured interview where blind to case/control status ❋

c) interview not blinded to case/control status

d) written self report or medical record only

e) no description

2) Same method of ascertainment for cases and controls

a) yes ❋

b) no

3) Non-Response rate

a) same rate for both groups ❋

b) non respondents described

c) rate different and no designation

Total 7/9

15. NEWCASTLE - OTTAWA QUALITY ASSESSMENT SCALE CASE CONTROL STUDIES – Vyas et al., 2016

Note: A study can be awarded a maximum of one star for each numbered item within the Selection and Exposure categories. A maximum of two stars can be given for Comparability.

Selection

1) Is the case definition adequate?

a) yes, with independent validation ❋

b) yes, eg record linkage or based on self reports

c) no description

2) Representativeness of the cases

a) consecutive or obviously representative series of cases ❋

b) potential for selection biases or not stated

3) Selection of Controls

a) community controls ❋

b) hospital controls

c) no description

4) Definition of Controls

a) no history of disease (**gastric cancer**) ❋

b) no description of source

Comparability

1) Comparability of cases and controls on the basis of the design or analysis
   a) study controls for **age and sex** (Select the most important factor.) ❋
   b) study controls for any additional factor ❋ (This criteria could be modified to indicate specific control for a second important factor.)

Exposure

1) Ascertainment of exposure
   a) secure record **(Serum 25(OH) vitamin D from laboratory measurements)** ❋
   b) structured interview where blind to case/control status ❋
   c) interview not blinded to case/control status
   d) written self report or medical record only
   e) no description
2) Same method of ascertainment for cases and controls
   a) yes ❋
   b) no
3) Non-Response rate
   a) same rate for both groups ❋
   b) non respondents described
   c) rate different and no designation

Total 6/9

16.NEWCASTLE - OTTAWA QUALITY ASSESSMENT SCALE CASE CONTROL STUDIES –

Vahid et al., 2018

Note: A study can be awarded a maximum of one star for each numbered item within the Selection and Exposure categories. A maximum of two stars can be given for Comparability.

Selection

1) Is the case definition adequate?
   a) yes, with independent validation ❋
   b) yes, eg record linkage or based on self reports
   c) no description
2) Representativeness of the cases
   a) consecutive or obviously representative series of cases ❋
   b) potential for selection biases or not stated

3) Selection of Controls
   a) community controls ❋
   b) hospital controls
   c) no description

4) Definition of Controls
   a) no history of disease (**gastric cancer**) ❋
   b) no description of source

Comparability

1) Comparability of cases and controls on the basis of the design or analysis
   a) study controls for **age and sex** (Select the most important factor.) ❋
   b) study controls for any additional factor (**BMI, smoking)** ❋ (This criteria could be modified to indicate specific control for a second important factor.)

Exposure

1) Ascertainment of exposure
   a) secure record (eg surgical records) ❋
   b) structured interview where blind to case/control status ❋
   c) interview not blinded to case/control status
   d) written self report or medical record only
   e) no description

2) Same method of ascertainment for cases and controls
   a) yes ❋
   b) no

3) Non-Response rate
   a) same rate for both groups ❋
   b) non respondents described
   c) rate different and no designation

Total: 8/9 stars

17. NEWCASTLE - OTTAWA QUALITY ASSESSMENT SCALE CASE CONTROL STUDIES – Yin., et al 2017

Note: A study can be awarded a maximum of one star for each numbered item within the Selection and Exposure categories. A maximum of two stars can be given for Comparability.

Selection

1) Is the case definition adequate?
  a) yes, with independent validation ❋
  b) yes, eg record linkage or based on self reports
  c) no description
2) Representativeness of the cases
  a) consecutive or obviously representative series of cases ❋
  b) potential for selection biases or not stated
3) Selection of Controls
  a) community controls ❋
  b) hospital controls
  c) no description
4) Definition of Controls
  a) no history of disease (**gastric cancer**) ❋
  b) no description of source

Comparability

1) Comparability of cases and controls on the basis of the design or analysis
  a) study controls for **age and sex** (Select the most important factor.) ❋
  b) study controls for any additional factor **(Smoking status and alcohol consumption)** ❋ (This criteria could be modified to indicate specific control for a second important factor.)

Exposure

1) Ascertainment of exposure
  a) secure record (**Genotyping performed using LDR**) ❋
  b) structured interview where blind to case/control status ❋
  c) interview not blinded to case/control status
  d) written self report or medical record only
  e) no description
2) Same method of ascertainment for cases and controls
  a) yes ❋
  b) no
3) Non-Response rate
  a) same rate for both groups ❋
  b) non respondents described
  c) rate different and no designation

Total 8/9 stars

18. NEWCASTLE - OTTAWA QUALITY ASSESSMENT SCALE COHORT STUDIES – Chen et

al., 2007

Note: A study can be awarded a maximum of one star for each numbered item within the Selection and Outcome categories. A maximum of two stars can be given for Comparability

Selection

1) Representativeness of the exposed cohort

a) truly representative of the average adult population of Linxian, China (describe) in the community ❋

b) somewhat representative of the average _______________ in the community ❋

c) selected group of users eg nurses, volunteers

d) no description of the derivation of the cohort

2) Selection of the non exposed cohort

a) drawn from the same community as the exposed cohort ❋

b) drawn from a different source

c) no description of the derivation of the non exposed cohort

3) Ascertainment of exposure

a) secure record **(Stored baseline serum samples analyzed for 25(OH)D concentration)** ❋

b) structured interview ❋

c) written self report

d) no description

4) Demonstration that outcome of interest was not present at start of study

a) yes ❋

b) no

Comparability

1) Comparability of cohorts on the basis of the design or analysis

a) study controls for **age and sex** (select the most important factor) ❋

b) study controls for any additional factor (smoking and alcohol consumption) ❋ (This criteria could be modified to indicate specific control for a second important factor.)

Outcome

1) Assessment of outcome

a) independent blind assessment ❋

b) record linkage ❋

c) self report

d) no description

2) Was follow-up long enough for outcomes to occur

a) yes **(> 5 years)** (select an adequate follow up period for outcome of interest) ❋

b) no

3) Adequacy of follow up of cohorts

a) complete follow up - all subjects accounted for ❋

b) subjects lost to follow up unlikely to introduce bias - small number lost - > ____ % (select an adequate %) follow up, or description provided of those lost) ❋

c) follow up rate < ____% (select an adequate %) and no description of those lost

d) no statement

Total: 9/9 stars

19. NEWCASTLE - OTTAWA QUALITY ASSESSMENT SCALE COHORT STUDIES – Abnet et al., 2010

Note: A study can be awarded a maximum of one star for each numbered item within the Selection and Outcome categories. A maximum of two stars can be given for Comparability

Selection

1) Representativeness of the exposed cohort

a) truly representative: **adults from the 8 prospective cohorts (1065 cases)** ❋

b) somewhat representative of the average ______________ in the community ❋

c) selected group of users eg nurses, volunteers

d) no description of the derivation of the cohort

2) Selection of the non exposed cohort

a) drawn from the same community as the exposed cohort ❋

b) drawn from a different source

c) no description of the derivation of the non exposed cohort

3) Ascertainment of exposure

a) secure record **(pre-diagnose serum 25(OH)D measurements from stored blood samples)** ❋

b) structured interview ❋

c) written self report

d) no description

4) Demonstration that outcome of interest was not present at start of study

a) yes ❋

b) no

Comparability

1) Comparability of cohorts on the basis of the design or analysis

a) study controls for **age and sex** (select the most important factor) ❋

b) study controls for any additional factor **(Smoking, alcohol, history of gastric surgery)** ❋ (This criteria could be modified to indicate specific control for a second important factor.)

Outcome

1) Assessment of outcome

a) independent blind assessment ❋
b) record linkage ❋
c) self report

d) no description

2) Was follow-up long enough for outcomes to occur
a) yes **(> 5 years)** (select an adequate follow up period for outcome of interest) ❋

b) no

3) Adequacy of follow up of cohorts
a) complete follow up - all subjects accounted for ❋
b) subjects lost to follow up unlikely to introduce bias - small number lost - > ____ % (select an adequate %) follow up, or description provided of those lost) ❋
c) follow up rate < ____% (select an adequate %) and no description of those lost
d) no statement

Total: 9/9 stars

20.NEWCASTLE - OTTAWA QUALITY ASSESSMENT SCALE COHORT STUDIES – Takasu et al., 2024

Note: A study can be awarded a maximum of one star for each numbered item within the Selection and Outcome categories. A maximum of two stars can be given for Comparability

Selection

1) Representativeness of the exposed cohort
a) truly representative: Large gastric cancer screening trial including adults from high-risk regions of Japan (1147 participants) in the community ❋
b) somewhat representative of the average ______________ in the community ❋
c) selected group of users eg nurses, volunteers

d) no description of the derivation of the cohort

2) Selection of the non exposed cohort
a) drawn from the same community as the exposed cohort ❋
b) drawn from a different source

c) no description of the derivation of the non exposed cohort

3) Ascertainment of exposure
a) secure record (**validated FFQs**) ❋
b) structured interview ❋
c) written self report

d) no description

4) <u>Demonstration that outcome of interest was not present at start of study</u>

a) yes ❋

b) no

Comparability

1) <u>Comparability of cohorts on the basis of the design or analysis</u>

a) study controls for **age and sex** (select the most important factor) ❋

b) study controls for any additional factor **(Smoking, H. pylori infection, pepsinogen levels)** ❋ (This criteria could be modified to indicate specific control for a second important factor.)

Outcome

1) <u>Assessment of outcome</u>

a) independent blind assessment ❋

b) record linkage ❋

c) self report

d) no description

2) <u>Was follow-up long enough for outcomes to occur</u>

a) yes **(6-year follow-up)** ❋

b) no

3) <u>Adequacy of follow up of cohorts</u>

a) complete follow up - all subjects accounted for ❋

b) subjects lost to follow up unlikely to introduce bias - small number lost - **around 5% of participants lacked follow-up** *(select an adequate %) follow up, or description provided of those lost)* ❋

c) follow up rate < ____% (select an adequate %) and no description of those lost

d) no statement

Total 9/9 stars

Appraisal tool for Cross-Sectional Studies (AXIS) – Kevin et al., 2021

No.	AXIS item	Yes	No	Don't know
**Introduction**				
1	Were the aims/objectives of the study clear?	X		
**Methods**				
2	Was the study design appropriate for the stated aim(s)?	X		
3	Was the sample size justified?	X		
4	Was the target/reference population clearly defined?	X		
5	Was the sample frame taken from an appropriate population base so that it closely represented the target/reference population?	X		
6	Was the selection process likely to select participants representative of the target population?	X		
7	Were measures undertaken to address and categorise non-responders?		X	
8	Were the risk factor and outcome variables measured appropriately?	X		
9	Were the risk factor and outcome variables measured correctly using validated instruments?	X		
10	Was statistical significance and/or precision clearly reported (e.g. p-values, CIs)?	X		

11	Were the methods sufficiently described to enable replication?	X		
**Results**				
12	Were the basic data adequately described?	X		
13	Does the response rate raise concerns about non-response bias?			X
14	If appropriate, was information about non-responders described?		X	
15	Were the results internally consistent?	X		
16	Were the results presented for all analyses described in the methods?	X		
**Discus-sion**				
17	Were the authors' discussions and conclusions justified by the results?	X		
18	Were the limitations of the study discussed?	X		
**Other**				
19	Were there any funding sources or conflicts of interest that may affect interpretation?	X		
20	Was ethical approval or informed consent obtained?	X		

Item no. 7 – No measures to address potential non-response bias were reported
Item no. 13 – The response rate was not reported
Item no. 14 – Characteristics or number of non-responders were not described

Appraisal tool for Cross-Sectional Studies (AXIS) – Kwak et al., 2020

**No.**	**AXIS item**	**Yes**	**No**	**Don't know**
**Introduc-tion**				
1	Were the aims/objectives of the study clear?	X		
**Methods**				
2	Was the study design appropriate for the stated aim(s)?	X		
3	Was the sample size justified?		X	
4	Was the target/reference population clearly defined?	X		
5	Was the sample frame taken from an appropriate population base so that it closely represented the target/reference population?	X		

6	Was the selection process likely to select participants representative of the target population?	X		
7	Were measures undertaken to address and categorise non-responders?		X	
8	Were the risk factor and outcome variables measured appropriately?	X		
9	Were the risk factor and outcome variables measured correctly using validated instruments?	X		
10	Was statistical significance and/or precision clearly reported (e.g. p-values, CIs)?	X		
11	Were the methods sufficiently described to enable replication?	X		
**Results**				
12	Were the basic data adequately described?	X		
13	Does the response rate raise concerns about non-response bias?			X
14	If appropriate, was information about non-responders described?		X	
15	Were the results internally consistent?	X		
16	Were the results presented for all analyses described in the methods?	X		
**Discussion**				
17	Were the authors' discussions and conclusions justified by the results?	X		
18	Were the limitations of the study discussed?	X		
**Other**				
19	Were there any funding sources or conflicts of interest that may affect interpretation?	X		
20	Was ethical approval or informed consent obtained?	X		

Item no. 3 – No formal sample size or power calculation was reported
Item no. 7 – No information was provided regarding non-responders or strategies to minimize non-response bias
Item no. 13 – The study was based on secondary analysis of the KHANES database; the analysed sample could not be assessed correctly
Item no. 14 – Information regarding characteristics of non-responders was not reported
