# Supplementary material for: Vitamin D and Gastric Cancer: A Systematic Review and Meta-Analysis
Source: Med Sci (Basel). 2026 Sep 11;14(5):562. doi: 10.3390/medsci14050562 (PMC13608993; doi:10.3390/medsci14050562)
Supplement: Supplementary file 1 [file medsci-14-00562-s001.zip › medsci-4532650 supplementary File S3.pdf]

Systematic Review

Vitamin D and Gastric Cancer: A Systematic Review and Meta-Analysis

Analysis	No. Studies	Pooled effect	95% CI	p	I^2	τ^2	Q (p)	95% PI
Serum 25(OH)D	4	MD -8.82	-18.15 to 0.51	0.057	96.68%	33.24	112.14 (<0.0001)	-29.41 to 11.76
Dietary vitamin D	5	OR 0.868	0.470–1.602	0.556	82.83%	0.183	20.39 (0.0004)	0.228–3.306
VDR FokI	5	OR 1.436	1.026–2.009	0.040	7.19%	0.0055	4.16 (0.385)	0.968–2.130

1. Serum 25(OH)D

Pooled MD = -8.82 ng/mL
95% CI: -18.15 to 0.51
p = 0.0573

Heterogeneity - high

τ^2 = 33.24
I^2 = 96.68%
Q = 112.14, df = 3
p < 0.0001

Prediction interval

95% PI: -29.41 to 11.76 ng/mL
The prediction interval is wide and includes 0.

Leave-one-out

Study omitted	MD	95% CI	I^2
Eom	-10.79	-24.02 to 2.45	96.25%
Durak	-10.12	-26.18 to 5.94	96.70%
Kevin	-6.54	-18.31 to 5.23	92.78%
Mushtaq	-7.78	-24.33 to 8.76	97.93%

I^2 remains between 92.78–97.93%. Kevin et al., exclusion reduces heterogeneity from 96.68% to 92.78%, but it is still high.

Influence analysis

Kevin et al. showed the greatest influence.

Sensitivity analysis: The pooled effect remained in the same direction after sequential exclusion of individual studies, with all 95% confidence intervals crossing the null value. Heterogeneity remained very high across leave-one-out analyses (I^2 = 92.78–97.93%). Kevin et al. showed the greatest influence on heterogeneity, reducing I^2 from 96.68% to 92.78% when omitted.

2. Dietary vitamin D

The model was fitted on the log(OR) scale, and the results are reported as ORs.

OR = 0.868
95% CI: 0.470–1.602
p = 0.5559

No statistically significant association was observed in the pooled analysis.

Heterogeneity – substantial

$\tau^2 = 0.1833$

$I^2 = 82.83\%$

Q = 20.39, df = 4

p = 0.0004

Prediction interval

95% PI on OR: 0.228–3.306

Large, includes 1.

Leave-one-out.

Study 95.	OR aproximativ	95% CI approximately	I^2
Pelucchi	0.786	0.354–1.748	85.13%
Nguyen	0.931	0.383–2.263	82.01%
Toorang	0.741	0.403–1.359	65.43%
Allehdan	0.964	0.471–1.973	84.32%
Chen	0.931	0.386–2.589	84.00%

None of the leave-one-out analyses became statistically significant.

Toorang et al. = important for heterogeneity; if we exclude it, we get I^2 decreasing from 82.83% to 65.43%.

Influence analysis

Toorang et al. showed the greatest influence, but this did not provide a reason for exclusion.

Sensitivity analysis: Sequential exclusion of individual studies did not materially alter the statistical significance of the pooled association, with all 95% confidence intervals crossing the null value. Heterogeneity remained substantial (I^2 = 65.43–85.13%), with Toorang et al. exerting the greatest influence and producing the largest reduction in I^2 when omitted.

3. VDR FokI polymorphism

Main result:

OR = 1.436

95% CI: 1.026–2.009

p = 0.0403

Statistically significant association.

Heterogeneity:

$\tau^2 = 0.0055$

$I^2 = 7.19\%$

Q = 4.16

p = 0.3851

Low heterogeneity was observed, and the Q test did not indicate statistically significant heterogeneity.

Prediction interval

95% PI: OR 0.968–2.130

PI includes 1, even if CI does not.

Leave-one-out.

Study 95.	OR aproximativ	95% CI approximately	I^2
Cong	1.363	0.837–2.216	25.53%

Durak	1.507	0.990–2.296	6.16%
Parsamanesh	1.554	1.064–2.271	0%
Hosseinkhani	1.431	0.878–2.333	27.58%
Qadir	1.308	0.919–1.862	0%

The pooled association remained statistically significant only after exclusion of Parsamanesh et al.; after exclusion of each of the other four studies, the 95% confidence interval crossed the null value.

Influence analysis

Qadir et al. has the biggest influence.

Sensitivity analysis: The pooled association remained statistically significant only after exclusion of Parsamanesh et al.; after exclusion of each of the other four studies, the 95% confidence interval crossed the null value. Heterogeneity remained low across leave-one-out analyses (I^2 = 0–27.58%). Qadir et al. showed the greatest influence in the influence analysis but was retained in the meta-analysis.

R Script

```
library(meta)
library(metafor)
#1.Serum 25(OH)D META-ANALYSIS
serum <- data.frame(
  study = c("Eom et al., 2018", "Durak et al., 2019", "Kevin et al., 2021", "Mushtaq et al., 2025"),
  n_cases = c(72,77,94,59),
  mean_cases = c(17.10, 11.00, 13.83, 20.15),
  sd_cases = c(8.90, 6.00, 5.97, 9.77),
  n_controls = c(91, 84, 94, 60),
  mean_controls = c(20.00, 16.00, 29.15, 32.21),
  sd_controls = c(6.50, 6.00, 4.13, 8.30))
str(serum)
summary(serum)
# Calculate mean differences for each study:
# MD = mean_cases - mean_controls
serum_es <- escalc(
  measure = "MD",
  m1i = mean_cases,
  sd1i = sd_cases,
  n1i = n_cases,
  m2i = mean_controls,
  sd2i = sd_controls,
  n2i = n_controls,
  data = serum)
serum_es
# Random-effects meta-analysis using REML
serum_model <- rma(
  yi = yi,
  vi = vi,
  data = serum_es,
  method = "REML",
  test = "knha")
serum_model
predict(serum_model)    # 95% prediction interval
```

```
confint(serum_model) # Confidence intervals for model parameters
# Leave-one-out sensitivity analysis
serum_loo <- leave1out(serum_model)
serum_loo
# The direction of the pooled effect remained unchanged across
# leave-one-out analyses. All 95% CIs crossed the null value.
# Heterogeneity remained very high (I2 = 92.78–97.93%).
# Kevin et al. had the greatest influence on heterogeneity.
#INFLUENCE ANAYSIS
influence_serum <- influence(serum_model)
influence_serum
# No study was excluded based on the influence analysis.
#Forestplot
forest(
  serum_model,
  slab = serum_es$study,
  xlab = "Mean Difference in serum 25(OH)D (ng/mL)",
  mlab = "Random-effects model (REML + HKSJ)",
  cex = 0.9)
forest(serum_model,
  # Study labels
  slab = serum_es$study,
  # X-axis
  xlab = "Mean Difference in serum 25(OH)D (ng/mL)",
  alim = c(-30, 10),
  at = seq(-30, 10, by = 5),
  xlim = c(-38, 20),
  # Study positions
  rows = c(4, 3, 2, 1),
  # Header
  header = c("Study", "MD [95% CI]"),
  # Overall effect
  mlab = "Overall effect",
  # Study appearance
  pch = 15,
  psize = 1.3,
  colout = "#1F4E79",
  # Overall diamond
  col = "#5B9BD5",
  border = "#1F4E79",
  # Make diamond taller
  efac = c(1, 3),
  # Text
  cex = 0.9,
  cex.lab = 0.95,
  cex.axis = 0.85,
  # Lines
  lwd = 1.2,
  # Numbers
  digits = 2)
# 2. DIETARY VITAMIN D META-ANALYSIS
```

```
dietary <- data.frame(
  study = c("Pelucchi et al., 2009", "Nguyen et al., 2022", "Toorang et al., 2022", "Al-lehdan et al., 2024", "Chen et al., 2025"),
  OR = c(1.33, 0.68, 1.59, 0.47, 0.67),
  lower_CI = c(0.80, 0.53, 1.07, 0.23, 0.48),
  upper_CI = c(2.21, 0.86, 2.36, 0.95, 0.92))
View(dietary)
str(dietary)
# Calculate effect sizes; for ORs, the analysis is performed on the log(OR) scale.
dietary$yi <- log(dietary$OR)
dietary$sei <- (log(dietary$upper_CI) - log(dietary$lower_CI)) / (2 * qnorm(0.975))
dietary$vi <- dietary$sei^2
dietary
# Random-effects model using REML
dietary_model <- rma(
  yi = yi,
  vi = vi,
  data = dietary,
  method = "REML",
  test = "knha")
dietary_model
# Transform the pooled estimate from the log(OR) scale to OR
exp(coef(dietary_model))
exp(c(
  dietary_model$b,
  dietary_model$ci.lb,
  dietary_model$ci.ub))
dietary_pred <- predict(dietary_model)
dietary_pred
exp(c(
  dietary_pred$pi.lb,
  dietary_pred$pi.ub))
# Leave-one-out sensitivity analysis
dietary_loo <- leave1out(dietary_model)
dietary_loo
# No study provided sufficient justification for exclusion based on the leave-one-out analysis.
# Toorang et al. had the greatest influence on heterogeneity, reducing I2 from 82.83% to 65.43% when omitted.
influence_dietary <- influence(dietary_model)
influence_dietary
# Toorang et al. showed the greatest influence, but this did not provide
# sufficient justification for exclusion.
# Forest plot
# atransf = exp because the model is fitted on the log(OR) scale
# and results are displayed as ORs.
forest(
  dietary_model,
  slab = dietary$study,
  # X-axis on the log(OR) scale
  alim = log(c(0.2, 4)),
```

```
    at = log(c(0.25, 0.5, 1, 2, 4)),
    xlim = c(-4, 3),
    rows = c(5, 4, 3, 2, 1),
    header = c("Study", "OR [95% CI]"),
    mlab = "Overall effect",
    pch = 15,
    psize = 1.3,
    colout = "#2E7D32",
    col ="#81C784",
    border = "#2E7D32",
    efac = c(1, 3),
    cex = 0.9,
    cex.lab = 0.95,
    cex.axis = 0.85,
    lwd = 1.2,
    digits = 2,
    # Show results as OR rather than log(OR)
    atransf = exp,
    xlab = "Odds Ratio for gastric cancer")
# 3. FOKI META-ANALYSIS
genetic <- data.frame(
    study = c(
        "Cong et al., 2015",
        "Durak et al., 2019",
        "Parsamanesh et al., 2019",
        "Hosseinkhani et al., 2021",
        "Qadir et al., 2023"),
    cases_exposed = c(145, 40, 67, 67, 99),
    cases_unexposed = c(42, 37, 36, 32, 44),
    controls_exposed = c(144, 42, 94, 60, 80),
    controls_unexposed = c(68, 42, 53, 40, 70))
View(genetic)
str(genetic)
# Calculate ORs from 2x2 contingency tables
genetic_es <- escalc(measure = "OR",
    ai = cases_exposed,
    bi = cases_unexposed,
    ci = controls_exposed,
    di = controls_unexposed,
    data = genetic)
genetic_es
# Random-effects model using REML
genetic_model <- rma(
    yi = yi,
    vi = vi,
    data = genetic_es,
    method = "REML",
    test = "knha")
genetic_model
# Low heterogeneity was observed.
exp(c(
```

```
    genetic_model$b,
    genetic_model$ci.lb,
    genetic_model$ci.ub))
genetic_pred <- predict(genetic_model)
genetic_pred
exp(c(
    genetic_pred$pi.lb,
    genetic_pred$pi.ub))
# Leave-one-out sensitivity analysis
genetic_loo <- leave1out(genetic_model)
genetic_loo
# The pooled association remained statistically significant only after exclusion
# of Parsamanesh et al.; after exclusion of each of the other four studies,
# the 95% confidence interval crossed the null value.
# No study was excluded from the meta-analysis, as all studies remained eligible.
influence_genetic <- influence(genetic_model)
influence_genetic
# Qadir et al. showed the greatest influence on the pooled estimate.
#Forest plot
forest(
    genetic_model,
    slab = genetic_es$study,
    alim = log(c(0.5, 3)),
    at = log(c(0.5, 1, 1.5, 2, 3)),
    xlim = c(-3.5, 3.5),
    rows = c(5, 4, 3, 2, 1),
    header = c("Study", "OR [95% CI]"),
    mlab = "Overall effect",
    pch = 15,
    psize = 1.3,
    colout = "#6A1B9A",
    col = "#BA68C8",
    border = "#6A1B9A",
    efac = c(1, 3),
    cex = 0.9,
    cex.lab = 0.95,
    cex.axis = 0.85,
    lwd = 1.2,
    digits = 2,
    atransf = exp,
    xlab = "Odds Ratio for gastric cancer")
```
